# Impact of human-associated *Escherichia coli* clonal groups in Antarctic pinnipeds: presence of ST73, ST95, ST141 and ST131

**DOI:** 10.1038/s41598-018-22943-0

**Published:** 2018-03-16

**Authors:** Azucena Mora, Francisco Javier García-Peña, María Pilar Alonso, Susana Pedraza-Diaz, Luis Miguel Ortega-Mora, Daniel Garcia-Parraga, Cecilia López, Susana Viso, Ghizlane Dahbi, Juan Marzoa, Martin J. Sergeant, Vanesa García, Jorge Blanco

**Affiliations:** 1 Laboratorio de Referencia de Escherichia coli (LREC), Departamento de Microbioloxía e Parasitoloxía, Facultade de Veterinaria, Universidade de Santiago de Compostela (USC), Lugo, Spain; 2 Laboratorio Central de Veterinaria de Algete, Ministerio de Agricultura y Pesca, Alimentación y Medio Ambiente, Ctra Madrid-Algete km 8, Madrid, Spain; 3 Servicio de Microbiología, Hospital Universitario Lucus Augusti (HULA), Lugo, Spain; 4 SALUVET, Animal Health Department, Faculty of Veterinary Sciences, Complutense University of Madrid, Madrid, Spain; 5 L’Oceanogràfic, Ciudad de las Artes y las Ciencias, Junta de Murs i Valls, Valencia, Spain; 6 Microbiology and Infection Unit, Warwick Medical School, University of Warwick, Coventry, United Kingdom; 7 Present Address: Environmental Toxicology, National Center for Environmental Health (CNSA), Institute of Health Carlos III, Madrid, Spain

## Abstract

There is growing concern about the spreading of human microorganisms in relatively untouched ecosystems such as the Antarctic region. For this reason, three pinniped species (*Leptonychotes weddellii*, *Mirounga leonina* and *Arctocephalus gazella*) from the west coast of the Antartic Peninsula were analysed for the presence of *Escherichia* spp. with the recovery of 158 *E*. *coli* and three *E*. *albertii* isolates. From those, 23 harboured different *eae* variants (α1, β1, β2, ε1, θ1, κ, ο), including a *bfpA*-positive isolate (O49:H10-A-ST206, *eae*-k) classified as typical enteropathogenic *E*. *coli*. Noteworthy, 62 of the 158 *E*. *coli* isolates (39.2%) exhibited the ExPEC status and 27 (17.1%) belonged to sequence types (ST) frequently occurring among urinary/bacteremia ExPEC clones: ST12, ST73, ST95, ST131 and ST141. We found similarities >85% within the PFGE-macrorrestriction profiles of pinniped and human clinic O2:H6-B2-ST141 and O16:H5/O25b:H4-B2-ST131 isolates. The *in silico* analysis of ST131 Cplx genomes from the three pinnipeds (five O25:H4-ST131/PST43-*fimH*22-virotype D; one O16:H5-ST131/PST506-*fimH*41; one O25:H4-ST6252/PST9-*fimH*22-virotype D1) identified IncF and IncI1 plasmids and revealed high core-genome similarities between pinniped and human isolates (*H*22 and *H*41 subclones). This is the first study to demonstrate the worrisome presence of human-associated *E*. *coli* clonal groups, including ST131, in Antarctic pinnipeds.

## Introduction

The Antarctic regions are regarded as protected areas with limited anthropogenic influence. However, there is growing concern about the impact of scientific and tourism activities which increase the risk of spreading human microorganisms in such relatively untouched ecosystems^1^. Marine mammals are considered as bio-indicators of possible environmental changes^2,3^ but most studies focused on the effect of untreated sewage discharges next to research stations, with few data about the presence of human-associated microorganisms within these animals^4^.

Although it is well-known that *Escherichia coli* is a common member of the intestine microbiota of warm-blooded vertebrates, recovery of *E*. *coli* seems to be low in wild marine mammals living in pristine environments when compared to captive animals or those close to locations with high anthropogenic exposure^5,6^. *Escherichia coli* is also a major intestinal and extraintestinal pathogen by means of successful combinations of virulence factors that have classically defined specific pathotypes^7^. Most extraintestinal *E*. *coli* infections are caused by isolates from phylogenetic group B2 which includes clinically significant subgroups such as ST73, ST131 or ST95^8^. ST131 has received special attention and much of its success is due to the spread of two fluoroquinolone-resistant subclones known as *H*30R/C1 and *H*30Rx/C2, frequently associated with human urinary tract infections and bacteriemia^9–11^. So far, the detection of ST131 in Antarctica has been twice reported from the isolation of water and/or marine sediments next to the research bases^4,12^.

The aims of the present study were i) to investigate the occurrence and characteristics of *Escherichia* spp. isolates in pinnipeds from the Antarctic regions ii) to assess the presence of high- risk clones in an ecological reserved region iii) to define possible sources of contamination and impact within this vulnerable ecosystem.

## Results

### O:H typing, phylogroups, pathotypes and ExPEC status

A total of 161 *Escherichia* spp. isolates recovered from Antarctic fur seals (80 isolates), Southern elephant seals (62 isolates) and Weddell seals (19 isolates) were phenotypically and molecularly characterized in this study. Of 63 different O:H antigen combinations, 10 accounted for 46.6% of the isolates: O11:H6 (five isolates), O73:HNM (five isolates), O15:H21 (six isolates), O51:H49 (six isolates), O25:H4 (seven isolates), O77:H45 (seven isolates), O120:H1 (seven isolates), ONT:H6 (eight isolates), ONT:H21 (10 isolates), O6:H1 (14 isolates). Phylotyping showed that the majority (84 isolates; 52.2%) belonged to phylogroup B2, followed by D (25; 15.5%), B1 (24; 14.9%), F (11; 6.8%), E (8; 5.0%) and A (6; 3.7%). Finally, three isolates (EC-25, EC-76 and EC-172b) that could not be assigned to any phylogroup were identified as *Escherichia albertii* (Supplementary Table S1).

The 161 isolates were analyzed by PCR for the presence of specific virulence factors (VF). None was positive for the *vt*_1_, *vt*_2_, *ipaH*, pcDV432, *eltA*, *est* or *stb* markers associated with verotoxigenic, enteroinvasive, enteroaggregative or enterotoxigenic pathotypes (Supplementary Table S1). However, 23 of the 161 (14.3%) isolates recovered from the three populations were carriers of the *eae* gene: Southern elephant seals (11 isolates, 17.7%), Antarctic fur seals (seven, 8.7%) and Weddell seals (five, 26.3%). These *eae*-positive isolates were identified as enteropathogenic *E*. *coli* (EPEC) (20 isolates) and *E*. *albertii* (three isolates), which belonged to 12 serotypes and harboured seven *eae* variants: α1 (six isolates), β1 (one isolate), β2 (two isolates), ε1 (three isolates), κ (seven isolates), θ1 (two isolates), and ο (two isolates). Additionally, the O49:H10 *eae*-κ isolate (EC-147) from an Antartic fur seal was *bfpA*-positive and therefore designed as typical enteropathogenic *E*. *coli* (tEPEC) (Table 1).

**Table 1:** Phenotypic and genotypic characterization of the 23 isolates (20 *E*. *coli* and three *E*. *albertii*) carriers of the *eae* gene.

Code	Serotype^a^	ST^b^	FG^c^	Clonotype^d^	Location	Origin of isolation	Virulence gene profile	*eae* typing	Resistance^e^
EC-3	O145:H25	STnew1	A	46-C	Byers Peninsula	Southern elephant seal	*fimH fimAv*_*MT78*_ *iutA iucD traT eae*	*eae* -epsilon1 (ε1)	
EC-78	O145:H25	STnew1	A	46-C	Avian Island	Southern elephant seal	*fimH iutA iucD traT eae*	*eae* -epsilon1 (ε1)	
EC-95	O145:H25	STnew1	A	46-C	King George Island	Southern elephant seal	*fimH fimAv*_*MT78*_ *iutA iucD traT eae*	*eae* -epsilon1 (ε1)	
EC-61	O8:HNM	STnew2	A	46-C	Avian Island	Southern elephant seal	*fimH fimAv*_*MT78*_ *iutA iucD traT eae*	*eae* -theta1 (θ1)	
EC-64	O8:HNM	STnew2	A	46-D	Avian Island	Antarctic fur seal	*fimH fimAv*_*MT78*_ *iutA iucD traT eae*	*eae* -theta1 (θ1)	
EC-147	**O49:H10**	**206**	**A**	7-E	Deception Island	Antarctic fur seal	*fimH iutA iucD traT eae bfp*	***eae*** **-kappa (k)**	
EC-136	O55,91:H11	29	B1	4–86	Deception Island	Antarctic fur seal	*fimH cdt3 traT malX eae*	*eae* -kappa (k)	
EC-137	O55,91:H11	29	B1	4–86	Deception Island	Weddell seal	*fimH cdt3 traT malX eae*	*eae* -kappa (k)	
EC-138	O55,91:H11	29	B1	4–86	Deception Island	Weddell seal	*fimH cdt3 traT malX eae*	*eae* -kappa (k)	
EC-88*	**O88:H25**	**328**	**B1**	23–31	King George Island	Antarctic fur seal	*fimH31 focG iutA iucD iroN traT eae*	***eae*** **-beta1 (β1)**	
EC-90	O33:HNM	28	B2	21–90	King George Island	Southern elephant seal	*fimH fimAv*_*MT78*_ *cdt1 iutA iucD traT malX usp eae*	*eae* -beta2 (β2)	
EC-91	O33:HNM	28	B2	21–90	King George Island	Southern elephant seal	*fimH fimAv*_*MT78*_ *cdt1 iutA iucD traT malX usp eae*	*eae* -beta2 (β2)	
EC-38	O51:HNM	STnew3	B2	568-B	Ronge Island	Weddell seal	*fimH cdt3 iutA iucD eae*	*eae* -kappa (k)	NAL
EC-52	O88:HNM	STnew4	B2	568–438	Avian Island	Weddell seal	*fimH cdt3 iutA iucD malX eae*	*eae* -kappa (k)	
EC-67	**O51:H49**	589	B2	39–135	Avian Island	Southern elephant seal	*fimH iutA iucD traT eae*	*eae* -alfa1 (α1)	
EC-82	**O51:H49**	589	B2	39–135	Avian Island	Southern elephant seal	*fimH iutA iucD traT eae*	*eae* -alfa1 (α1)	
EC-134	**O51:H49**	589	B2	39–135	Deception Island	Antarctic fur seal	*fimH iutA iucD traT eae*	*eae* -alfa1 (α1)	
EC-148	**O51:H49**	589	B2	39–135	Deception Island	Antarctic fur seal	*fimH iutA iucD traT tsh eae*	*eae* -alfa1 (α1)	
EC-156	**O51:H49**	589	B2	39–135	Deception Island	Weddell seal	*fimH iutA iucD traT eae*	*eae* -alfa1 (α1)	
EC-165	**O51:H49**	589	B2	39–135	Deception Island	Antarctic fur seal	*fimH iutA iucD traT eae*	*eae* -alfa1 (α1)	
EC-76	O84:HNM	2087	†	324-A	Avian Island	Southern elephant seal	*fimH fimAv*_*MT78*_ *cdt3 iutA iucD traT eae*	*eae* -omicron (o)	
EC-25	ONT:HNM	2087	†	324-A	Hannah Point	Southern elephant seal	*fimH cdt3 iutA iucD traT eae*	*eae* -omicron (o)	CEF
EC-172b	O128:HNM	STnew5	†	393–424	Deception Island	Southern elephant seal	*fimH cdt3 iutA iucD traT ibeA eae*	*eae* -kappa (k)	

^a^O antigen: nontypeable isolates were designated as ONT; H antigen: HNM for nonmotile isolates and HNT for those which did not react with any antisera.

^b^STs according to the Achtman scheme^14^; eight isolates showed new STs designated in this study as STnew1, STnew2, STnew3, STnew4 and STnew5.

^c^Phylogroups established by PCR according to the Clermont *et al*.^56^ scheme. †*Escherichia albertii* isolates.

^d^Clonotypes (*fumC* – *fimH* alleles)^36^; nine isolates showed new *fimH* alleles designated as A, B, C, D and E.

^e^Antimicrobial susceptibility was tested using the commercial broth microdilution minimum inhibitory concentration (MIC); the MICs were interpreted according to standard break points (CLSI, 2016); CEF: cefalotin, NAL: nalidixic acid.

*EC-88 positive for the ExPEC status.

In bold: serotypes or combinations of serotype/phylogroup/ST/intimin reported in human diarrheagenic EPEC.

The PCR-screening of 20 extraintestinal VF showed that all 161 isolates carried one to 13 genes (Supplementary Table S1). Furthermore, 62 of the 158 (39.2%) *E*. *coli* isolates were positive for two or more of the five markers *papC*, *sfa/focDE*, *afa/draBC*, *kpsM II* and *iutA* which define ExPEC status^13^, with similar prevalence within the pinniped populations (26.3% of Weddell seals, 37.3% of Southern elephant seals and 43.8% of Antarctic fur seals). Of the 62 ExPEC isolates, more than 50% were positive for *fimH* (100%), *sfa/focDE* (66.1%), *iutA* (62.9%), *iucD* (58.1%), *iroN* (64.5%), *kpsM II* (82.3%), *traT* (71.0%), *malX* (93.5%) and *usp* (79.0%) (Supplementary Table S2). Interestingly, one of the 62 ExPEC (EC-88) also harboured the *eae* gene (Table 1). Significant differences were observed between the 62 ExPEC (VF range of 5–13) and the 96 non-ExPEC isolates (VF range of 1–9) regarding the phylogroup distribution (1.6% *vs* 24.0% of B1, 85.5% *vs* 32.3% of B2, 0% *vs* 26.0% of D, respectively) (*P* < 0.001 for all comparisons).

### STs, virotypes, clonotypes and PFGE macrorrestriction profiles

**Figure 1:**
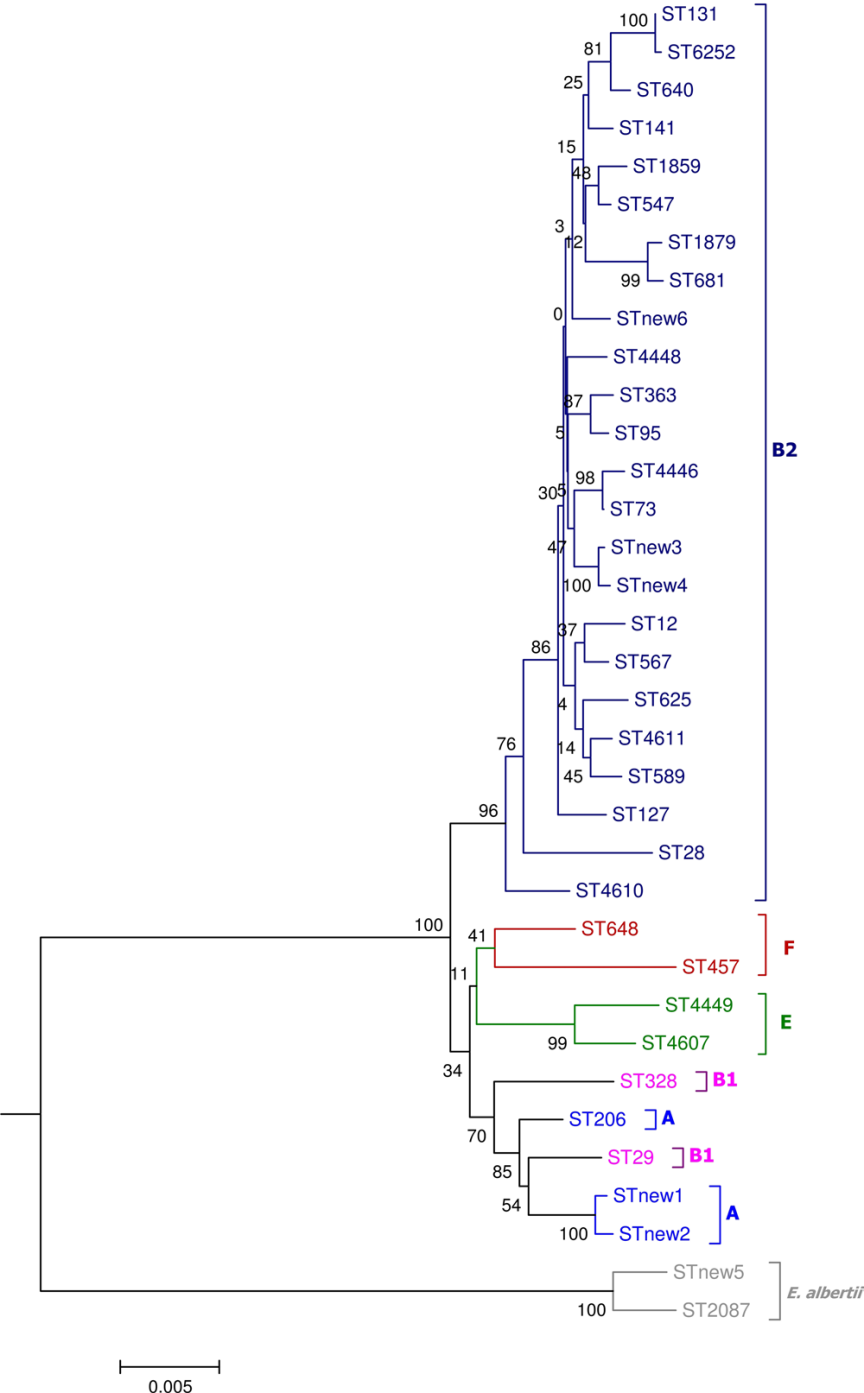
Phylogenetic tree based on concatenated sequences of the seven housekeeping genes from the MLST Achtman scheme by the Neighbor-Joining method using MEGA6: numbers on the tree indicate bootstrap values calculated for 1,000 replicates.

Eighty-five of the 161 isolates were further characterized by MLST and clonotyping including those identified as EPEC and/or ExPEC pathotypes and the three *E*. *albertii*. In total, 35 STs were established according to the Achtman scheme^14^, with 13 STs determined for first time in this study. Seven of those 13 new STs got numerical designations (ST4446, ST4448, ST4449, ST4607, ST4610, ST4611, ST6252) since they were performed before the requirement of WGS for new ST assignments, and the remaining six new allelic combinations were named here as STnew1 to STnew6. The Neighbor-Joining tree based on the concatenated sequences showed the phylogenetic relationship among the STs, consistently distributed in relation to their phylogroup and species (Fig. 1; Supplementary Table S3). The most prevalent STs were ST73 (14 isolates), ST1859 (eight isolates), ST131 (six isolates), ST589 (six isolates) and ST457 (five isolates).

Seven isolates belonged to the sequence type complex 131 (ST131 Cplx) and were further differentiated into three STs according to the Pasteur Institute scheme^15^ (PSTs): PST43 detected in five O25b:H4-ST131 isolates, PST9 in one O25b:H4-ST6252 and PST506 in one O16:H5-ST131 isolate. Besides, the virotype of these isolates was established based on the scheme of Dahbi *et al*.^16^ as virotype D1 (isolate EC-77), virotype D2 (isolates EC-23 and EC-24), virotype D-not typeable (EC-114, EC-115 and EC-160), while the virulence profile of the O16:H5 isolate (EC-152) did not match any of the 12 virotypes included in the scheme (Supplementary Table S1).

**Figure 2:**
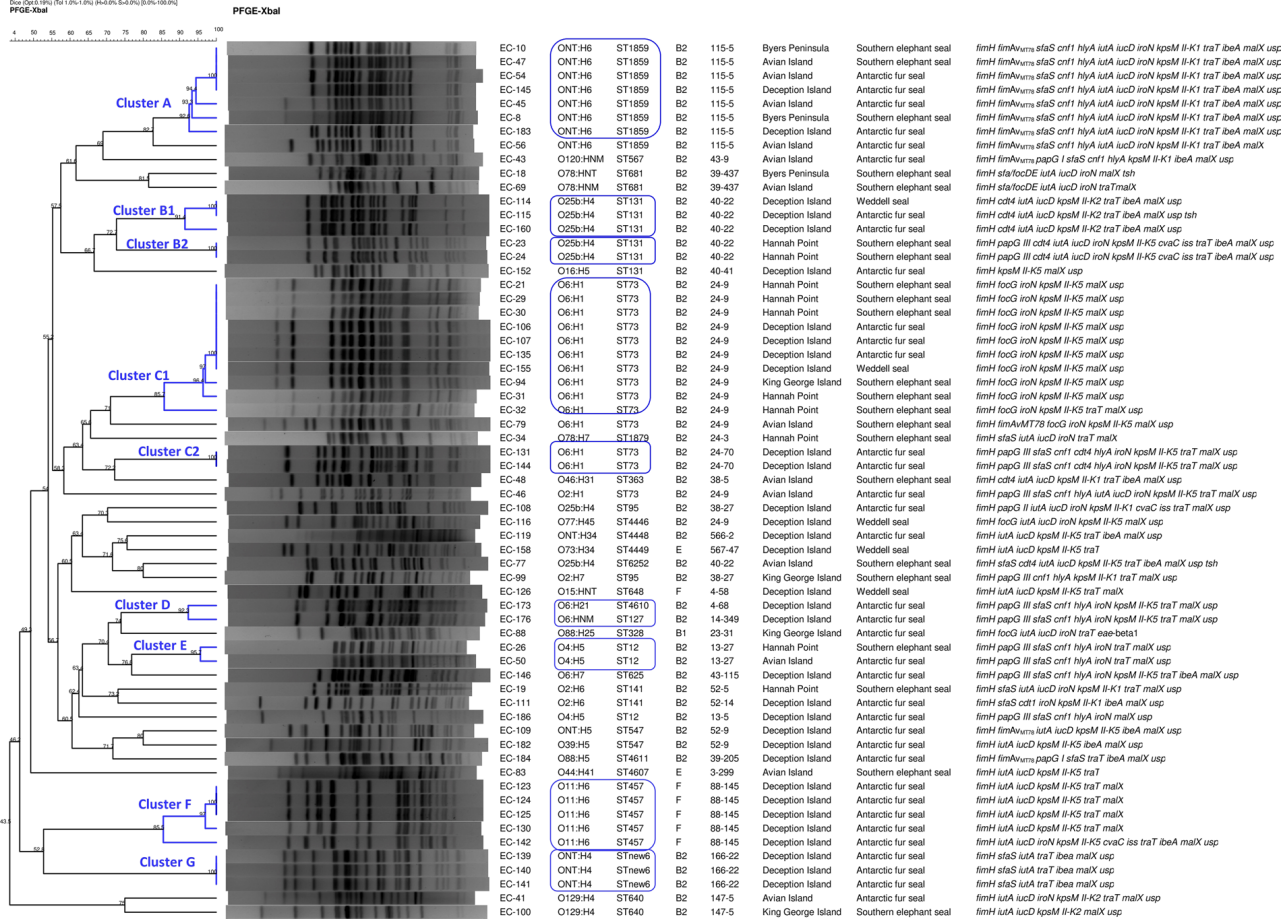
Dendrogram of the *XbaI* macrorestriction profiles of the 63 ExPEC and one non-ExPEC (O16:H5-B2-ST131): association between isolation code, serotype, ST, clonotype (*fumC*-*fimH* alleles), sampling location, origin of isolation and virulence-gene profile is indicated on the right.

Clonotyping revealed 11 different clonotypes (CH) among the 23 *eae*-positive isolates, with five new *fimH* alleles in the analysis of the 489-nucleotide (nt) internal fragment designated in this study as *fimH*_A to E (Table 1; Supplementary Figure S1). The new allele *fimH_*A identified in two *E*. *albertii* (EC-25 and EC-76) showed 99.39% of identity with *fimH*424 with three nt changes and one aminoacidic change (threonine for isoleucine). The *fimH*_B allele identified in one *E*. *coli* isolate (EC-38) showed 99.80% of identity with *fimH*438, with one nt and one aminoacidic change (alanine for threonine). Interestingly, *fimH*_C, *fimH*_D and *fimH*_E were four, three and one nt variants of *fimH*41, respectively, but only *fimH*_E gave an aminoacidic change (asparagine for aspartic acid). Further 25 different clonotypes were determined among the ExPEC isolates, whose genetic relatedness and diversity were additionally analyzed by *XbaI* macrorestriction followed by pulsed-field electrophoresis (PFGE) (Fig. 2). In this comparison, an O16:H5-B2-ST131 isolate negative for the ExPEC status was also included. Thus, the 63 isolates grouped in the dendrogram in relation to their serotype/phylogroup/ST, with nine clusters of identity ≥ 85% (Clusters A to G) including the main clonal groups.

**Figure 3:**
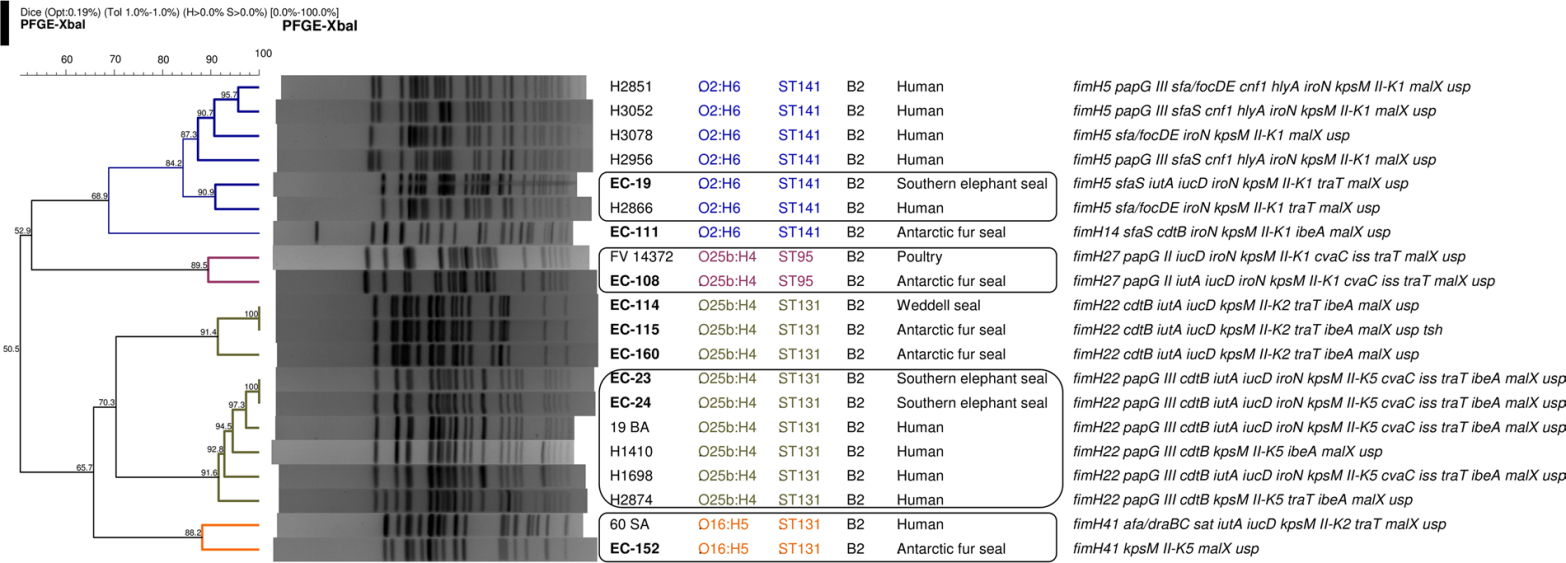
Dendrogram of the *XbaI* macrorestriction profiles of pinniped ExPEC belonging to the clonal groups ST95, ST141 and ST131 compared with clinic isolates: highlighted those of different origins showing >85% similarity.

We compared the pinniped isolates of the main B2 clonal groups with *E*. *coli* clinical isolates (animal and human) of our collections. As a result, we found high similarities (>85%) (Fig. 3) between the PFGE macrorestriction profiles of O2:H6-B2-ST141 *fimH*5 (one Southern elephant seal and one human isolates), O25b:H4-B2-ST95 *fimH*27 (one Antarctic fur seal and one poultry isolates), O25b:H4-B2-ST131 *fimH*22 (two Southern elephant seal and four human isolates) and O16:H5-B2-ST131 *fimH*41 (one Southern elephant seal and one human isolates).

### Resistance characterization

The antimicrobial susceptibility test using MIC panels including 19 antibiotics determined resistance values in six (3.7%) out of 161 isolates tested to ampicillin (two isolates), cefalotin (three isolates) and nalidixic acid (two isolates). Additionally, intermediate MIC values to ampicillin and cefalotin were detected in one and 14 isolates, respectively. By PCR, the 161 isolates were negative for *bla*_SHV_ and *bla*_CTX-M_, and only two (EC-23 and EC-24) gave positive result for *bla*_TEM_ whose sequence was identified as TEM-1.

### Whole genome sequencing of ST131Cplx isolates

Whole genome sequencing was performed for six of the seven ST131 Cplx isolates obtained from the three pinniped species (EC-23, EC-24 and EC-77 from Southern elephant seals; EC-114 from a Weddell seal; EC-152 and EC-160 from Antarctic fur seals). The draft genomes of the six isolates yielded 63 to 327 contigs larger ≥ 200 bp, with assembly sizes ranging from 4.8 Mb (EC-152) to 5.5 Mb (EC-77) (average of 5.1 Mb). The *in silico* determination of O/H antigens and STs (7 gene scheme) predicted by EnteroBase (https://enterobase.warwick.ac.uk/) was in agreement with the experimental data, with the exception of *adk* allele EC-24 that could not be predicted. The resistome of the draft genome, analysed using ResFinder showed the presence of antimicrobial resistance determinants *bla*_TEM-1C_ and *tet*(*A*) in EC-23, EC-24 and EC-77, while PlasmidFinder allowed the detection of plasmid replicons IncF (five isolates) and IncI1 (two isolates) (Table 2).

**Table 2:** O/H, ST, plasmid and resistance profiles of six ST131 Cplx *E*. *coli* pinniped and four human isolates based on WGS analysis.

Isolate code	Enterobase ID^a^	O^a^	H^a^	ST^a^	cgST^a^	wgST^a^	rST^a^	Sequences^b^	Coverage^c^	Identity (%)	Inc group (pMLST)^d^	Resistance genes^e^
EC-23	AM_LREC-53	O25	H4	131	30788	30659	1500	FII_1_AY458016 (RNAI-FII)	1-261/261	100.00	IncF (F2:A-:B1)	*bla*_TEM-1C_ *; tet(A)*
								FIB_1_AP001918 (*repFIB*)	1-682/682	98.39		
EC-24	AM_LREC-54	O25	H4	ND†	30793	30652	1500	FII_1_AY458016 (RNAI-FII)	1-261/261	100.00	IncF (F2:A-:B1)	*bla*_TEM-1C_ *; tet(A)*
								FIB_1_AP001918 (*repFIB*)	1-682/682	98.39		
EC-77	AM_LREC-55	O25	H4	6252	30794	30661	1503	FII_1_AY458016 (RNAI-FII)	1-261/261	95.79	IncF (F18:A-:B20*)	*bla*_TEM-1C_ *; tet(A)*
								FII(29)_pUTI89_CP003035 (*repFII*)	1-259/259	98.46		
								FIB_1_AP001918 (*repFIB*)	1-682/682	96.77		
								FIC_1_AP001918 (*repFIC*)	1-499/499	95.59		
EC-114	AM_LREC-56	O25	H4	131	21190	30689	1503	FIB_1_AP001918 (*repFIB*)	1-499/499	95.59	IncF (F18:A-:B20) +	None
								FIC_1_AP001918 (*repFIC*)	1-682/682	96.92	IncI1 (unknown ST)	
								I1_1_Alpha_AP005147 (RNAI-I1)	1-142/142	97.18		
EC-160	AM_LREC-57	O25	H4	131	30821	30697	1831	FIB_1_AP001918 (*repFIB*)	1-682/682	96.92	IncF (F18:A-:B20)	None
								FIC_1_AP001918 (*repFIC*)	1-499/499	95.59		
EC-152	AM_LREC-58	O16	H5	131	21179	30694	1503	I1_1_Alpha_AP005147 (RNAI-I1)	1-134/142	97.01	IncI1 (unknown ST)	None
19 BA	AM_LREC-3	O25	H4	131	30799	30663	ND	FIB_1_AP001918 (*repFIB*)	1-682/682	98.39	IncF (F-:A-:B1)	*bla*_TEM-1C_
H1410	AM_LREC-40	O25	H4	131	21188	30715	1500	X1_4_JN935898 (*repA*)	1-377/377	99.20	IncX1	None
H1698	AM_LREC-41	O25	H4	131	30841	30728	1500	FII(pSFO)_AF401292 (repFII)	1-258/258	95.74	IncF (F3*:A-:B-)	None
60 SA	AM_LREC-45	O16	H5	131	30848	30731	1503	FII(pRSB107)_1_AJ851089 (*repFII*)	1-261/261	100.00	IncF (F1:A1:B1) + IncQ1	*bla*_TEM-1B_; *strA*; *strB*; *aph*(3′)-Ia; *mph(A); catA1; sul2; tet(B); dfrA14*

^a^Information from Enterobase (https://enterobase.warwick.ac.uk/species/index/ecoli): Identification (ID); O/H antigens and STs based on the 7 gene (ST), core genome (cgST), whole genome (wgST) and RNA MLST schemes predicted by Enterobase. †adk allele of EC-24 could not be predicted (ND), however, the remaining alleles matched those of ST131.

^b,c^In silico determination using PlasmidFinder 1.3 (https://cge.cbs.dtu.dk/services/PlasmidFinder/): ^b^Locus targeted and GenBank accession number of the reference sequence; ^c^Number of query nucleotides found in the draft genome compared to the reference gene sequence deposited in the PlasmidFinder databases.

^d^Plasmid STs identified using pMLST 1.4 (https://cge.cbs.dtu.dk/services/pMLST/).

^e^Resistome of the draft genome determined using ResFinder 2.1 (https://cge.cbs.dtu.dk/services/ResFinder/).

**Figure 4:**
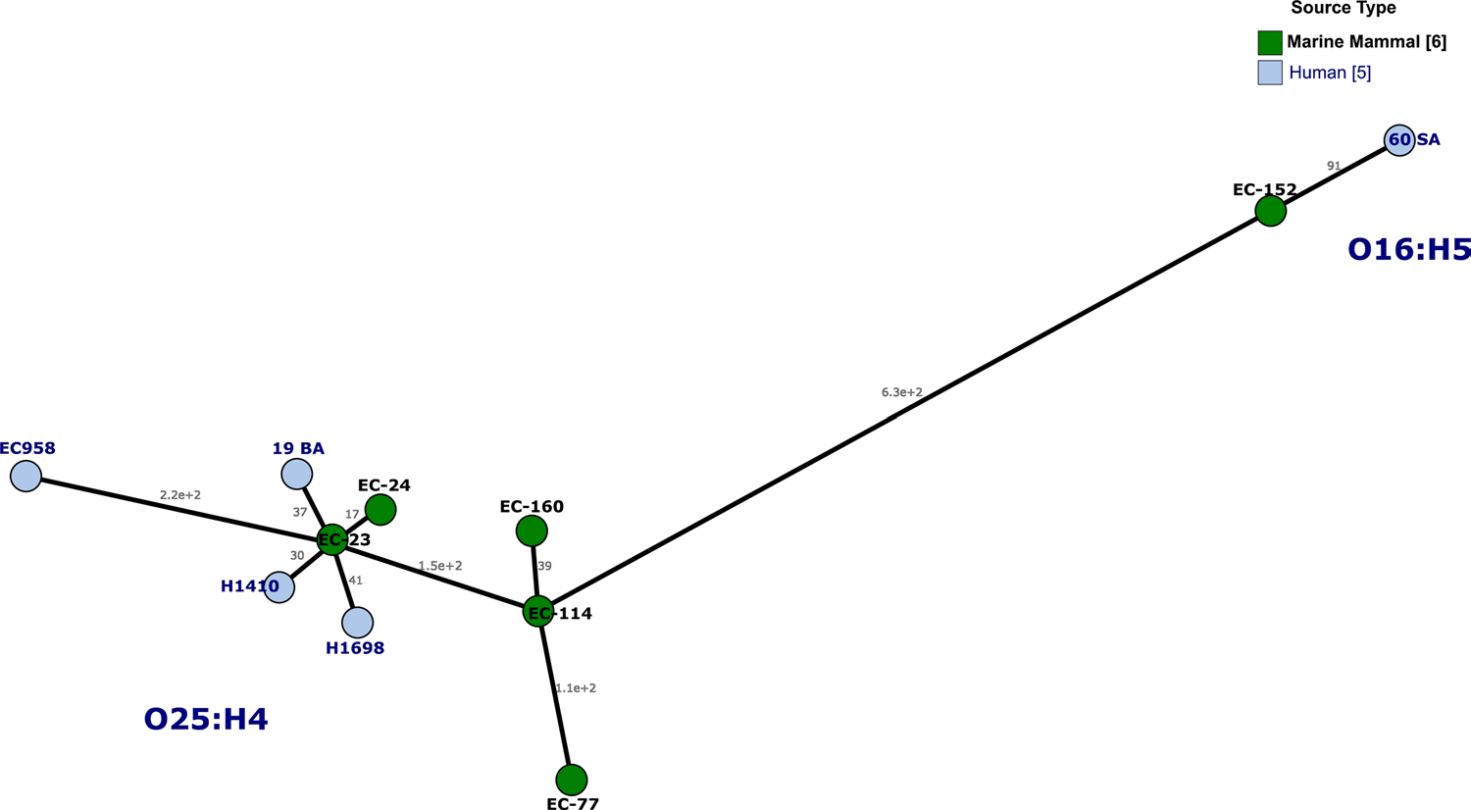
Minimum spanning tree based on the core genome MLST (cgMLST) using the MSTreeV2 tool from Enterobase: comparison of ST131 genomes (six from pinniped, four from human clinic isolates and the reference genome EC958 also obtained from a human isolate).

By genome comparison, we further investigated the relatedness between the six pinniped ST131 Cplx isolates and the four ST131 human clinic isolates with highly similar macrorrestriction profiles shown in Fig. 3, together with the ST131 reference genome EC958, all of them assembled in Enterobase (https://enterobase.warwick.ac.uk/). In the Minimum Spanning Tree of the core genome analysis based on the cgMLST scheme from Enterobase, the pinipped isolates EC-23 and EC-24 showed high genetic relatedness (58 and 147 differences, respectively) compared to human H1698 human isolate (all of subclone *H*22); likewise, the pinniped isolate EC-152 showed less than100 differences compared to human 60SA isolates (both of suclone *H*41) (Fig. 4). Five of the six WGS ST131 pinniped isolates carried contigs with IncF plasmid replicons of types F2:A-:B1 (predicted in EC-23 and EC-24) and F18:A-:B20 (predicted in EC-77, EC-114 and EC-160) (Table 2).

## Discussion

In this study, we analysed a collection of 161 *Escherichia* spp. isolates obtained from three Antarctic pinniped species sampled in six locations with different human activity along the west coast of the Antarctic Peninsula. While none was positive for the verotoxigenic, enteroinvasive, enteroaggregative or enterotoxigenic virulence markers, *eae*-positive isolates were identified from 26.3% of the Weddell seals, 17.7% of Southern elephant seals and 8.7% of the Antarctic fur seals. The EPEC pathotype is characterised by the presence of the locus of the enterocyte effacement (LEE) containing the *eae* gene and do not produce verotoxins^17^. EPEC isolates can be further classified into typical (tEPEC) and atypical (aEPEC) EPEC on the basis of the presence (tEPEC) or absence (aEPEC) of the EPEC adherence factor (EAF) plasmid, which harbours the *bfp* operon encoding the bundle-forming pilus (BFP)^17,18^. The tEPEC is a major cause of diarrhoeal disease among children in developing countries, being humans the major natural reservoir for tEPEC which has been rarely reported from animals^19–21^, while aEPEC has been widely isolated from both humans and animals^21,22^. *E*. *albertii* is an emerging pathogen identified as a causative agent in human gastroenteritis. *E*. *albertii* frequently carries the *eae* gene causing it to be mistakenly identified as EPEC or Enterohemorragic *E*. *coli* (EHEC), and also typically possesses the II/III/V subtype group *cdtB* gene^23^.

The 23 *eae*-positive pinniped isolates (19 aEPEC, one tEPEC and three *E*. *albertii*) belonged to 12 serotypes and 11 STs, some of them previously reported in human diarrheagenic aEPEC (namely, serotypes O88:H25 and O51:H49; STs 28, 29, 328 and 589) but also in other sources^24,25^ (Table 1). O51:H49-B2-ST589 (*eae*-α1), the most prevalent clonal group within the pinniped EPEC isolates, was also reported in freshwater samples on the English Channel coast^26^, and the ST589 in aEPEC human diarrheagenic and animal isolates in China^24^. Of note is the wide host range of clonal group O88:H25-B1-ST328 *eae*-β1, including humans, lambs and even shellfish^24–26^, determined in the pinniped isolate EC-88 positive for the ExPEC status and for the F1C fimbriae *focG* gene associated to urinary strains. This heterogenic virulence capability was previously described by Dutta *et al*.^27^ in an ST328 aEPEC (*eae*-β) isolate from a child with acute diarrhea, which also harboured the *elt* gene in a conjugative plasmid (IncFII) together with the P fimbriae *papG* and *papC* genes. In our study, the 23 *eae*-positive isolates carried extraintestinal VF (range 5–9) conforming to a hybrid pathotype. Likewise, the three pinniped *E*. *albertii* isolates not only carried the reported *eae* and *cdtB* genes^23^, but also other associated with ExPEC: *fimAv*_*MT78*_, *iutA*, *iucD*, *traT* and *ibeA*. Furthermore, one of the 23 *eae*-positive isolates (EC-147) was *bfpA*-positive (tEPEC) and belonged to the clonal group O49:H10-A-ST206 which is periodically detected in patients with diarrhea attending the hospital of our city (Supplementary Figure S2). Recently, Alonso *et al*.^28^ first reported this tEPEC clonal group from a wild boar, suggesting the acquisition from human sources. The surprising detection of this tEPEC from a marine mammal would need further investigation to explain its origin.

The ExPEC status was determined in 62 (39.2%) of the 158 *E*. *coli* recovered from the three pinniped species within different locations (16 of the 44 *E*. *coli* from rarely visited locations, four of the 17 from moderate visited locations, 42 of the 97 from highly visited locations), without significant difference in presence between rarely visited locations *vs* moderate plus highly visited (36.4% *vs* 40.3%) (*P* > 0.05).

ExPEC is a heterogeneous group, defined by isolation from infections outside the intestinal tract since no core set of virulence factors can unambiguously distinguish the ExPEC subgroups. Different researchers tried to elucidate this fact studying the phylogeny of the isolates, virulence traits and host factors^29^. In our study, the prevalence of certain extraintestinal VF was unexpectedly high and significantly associated to the 62 ExPEC isolates: *papC*, *papG III*, *sfaS*, *focG*, *cnf1*, *hlyA*, *iutA*, *iucD*, *iroN*, *kpsM II-K5*, *neuC* (K1), *traT*, *ibeA*, *malX* and *usp* (*P* < 0.001 for all comparisons). Many of those were previously correlated with urinary isolates together with the phylogenetic group B2^30,31^. Here, the phylogroup B2 accounted for more than 50% of the Antarctic collection and 85.5% of the 62 ExPEC isolates, including 19 B2 clonal groups repeatedly implicated in human urinary tract infections and bacteremia worldwide^8,32–34^, such as ST73 (14 isolates O6:H1/O2:H1-B2-ST73), ST131 (five isolates O25b:H4-B2-ST131), ST12 (three isolates O4:H5-B2-ST12), ST141 (two isolates O2:H6-B2-ST141), ST95 (two isolates O25b:H4/O2:H7-B2-ST95), that additionally showed clonotypes found within clinical isolates^35–37^ (Fig. 2). Another minor clonal groups from the pinniped collection reported in clinic extraintestinal isolates were: ST363-B2 (CH38–5), ST648-F (CH4-58); ST328-B1 (CH23-31), ST457-F (CH88-145); ST640-B2 (CH147-5)^37^. Previous studies on commensal *E*. *coli* isolated from birds, non-human mammals and humans found that the prevalence of the phylogenetic groups was clearly different with predominance of D/B1, A/B1 and A/B2, respectively. Although the isolates from 323 mammals did not encompassed any from marine animals, the phylogroup B2 was clearly the less prevalent of this group^38^. Nevertheless, a recent study on 40 *E*. *coli* recovered from fruit bats, in areas with very low human population density of the Republic of Congo, reported a B2 prevalence of 46.2%, with detection of EPEC and ExPEC pathotypes. The authors stated that known human isolates might be present in wild animals as part of the naïve *E*. *coli* population more frequently as originally assumed^39^.

Power *et al*.^4^ also identified a high prevalence of phylogroup B2 (44.5%) among 353 Antarctic *E*. *coli* isolates with presence of STs 12, 73, 95, 127, 141 and 131; however, most were seawater and sediment isolates, and only ST95 was detected in isolates from three Weddell seals. The ST95 is associated with a highly pathogenic group of ExPEC belonging to different serotypes such as O1:H7/HNM, O18:H7, or O45:H7/HNM frequently implicated in neonatal meningitis, urinary tract infections and septicemia in humans, but also in avian pathology^40,41^. We found two ST95 isolates (O2:H7 and O25b:H4) in this study. It is noteworthy the detection of an O25b:H4-B2-ST95 (*fimH*27) isolate since this serotype is usually associated to B2-ST131, although it can also be found within other clonal groups such as O25b:H4-D-ST69 first described in raw sewage and river water samples in Barcelona^42^. Interestingly, we found high similarity (89.9%) between the macrorrestriction/virulence profiles of one pinniped and one chicken O25b:H4-B2-ST95 isolates (Fig. 3).

As far as we know, the ST131 had not been previously determined in isolates from Antarctic animals, even though CTX-M-15-producing ST131 isolates were recovered in 2011 from water samples close to research bases on the Antarctic Peninsula and King George Island^12^. ST131 is a recently emerged and globally disseminated multidrug-resistant clone associated with human urinary tract and bloodstream infections, but rarely reported among animal ExPEC^43^. Genomic studies on the ST131 clonal evolution determined that ST131 first acquired genes linked to an increased capacity to cause human infection, and then gained an arsenal of resistance genes to diver in different clades: clade A associated mostly with *fimH*41, clade B associated with *fimH*22 and precursor of clade C, which is associated with *fimH*30. Recently, two subclades were identified within clade C: C1 or *H*30R (associated with fluoroquinolone-resistance) and C2 or *H*30-Rx (associated with CTX-M-15). These two subclades are currently recognized as the *E*. *coli* lineages most likely to be responsible for human extraintestinal infections^44^, although wide longitudinal studies in Spain^16^ and England^34^ determined that all of the ST131 clades were present during the whole study period, which might indicate that the entire ST131 clonal group is successful and not just subclades C1 and C2^34^. Here, we report the presence of ST131 Cplx in the three pinniped species from different locations, accounting for 4.3% of the 161 isolates. According to their *fimH* alleles, six isolates were of clade B (*H*22), one of clade A (*H*41) and none belonged to the C (*H*30) clade. The detection of O16:H5-ST131 subclone was especially unexpected since it is globally much less prevalent than O25b:H4-ST131, consistently accounting for approximately 1 to 5% of the *E*. *coli* clinical isolates overall^16,45^. All six *fimH*22 isolates showed virotype D in agreement with previous reports for this clade^16,46^.

Importantly, none of the sampled pinnipeds was positive for ESBL, and only six isolates exhibited resistance to ampicillin, cefalotin or nalidixic acid. Resistance within the classical ExPEC such as ST73 or ST95 has been unfrequently detected. However, emerging clonal groups such as ST131 or ST648 are known by their essential role in the global increase of antibiotic resistances^34,47,48^. Hernández *et al*.^12^ first reported ESBL-producing *E*. *coli* on the Antarctic region, specifically *bla*_CTX-M-1_ and *bla*_CTX-M-15_ genotypes (including ST131 isolates). Nevertheless, those clearly human-associated Enterobacteriaceae were not detected among 400 fresh penguin fecal droppings sampled near the bases which could indicate, according to the authors, infrequent interactions between penguins and humans or a less opportunistic diet compared with other feeders such as wild birds^49^.

Epidemic resistance plasmids belonging to IncF with divergent replicon types (such as FIA, FIB, and FII) have the ability to acquire resistance genes and then rapidly disseminate among Enterobacteriaceae^50^. Moreover, it has been postulated that the F-type plasmids shaped the evolution of the *H*30 subclone and specifically, an F1:A2:B20 plasmid would be strongly associated with the *H*30R/C1 clade, and an F2:A1:B- plasmid with the *H*30Rx/C2 clade^51^. Although the F2:A1 allele combination is dominating among *H*30Rx isolates, it also occurs among *H*22 and *H*41, and the F1:A2:B20 allele among *H*30Rx and *H*41 isolates. However, F18, F29, B1 and B10 were the most common replicon alleles among ancestral isolates according to the findings of Johnson *et al*.^51^. As a consequence, the IncF-type plasmids determined in the pinniped ST131 isolates might derived from ancestral lineages.

## Conclusions

The present study provides evidence for Antarctic marine mammals carrying potentially pathogenic *E*. *coli* pathotypes which are frequently involved in enteropathogenic and extraintestinal human diseases. We report for first time the presence of ST131 Cplx recovered from three pinniped species sampled in different locations. The phylogenetic distribution, virulence profiles, STs and genomic traits would indicate possible ancestral sublineages of current clinic clonal groups. The challenge now is to answer how and when these isolates became part of the commensal microbiota of these marine mammals. A possible explanation might be geographic mobility of these animals, contacting areas of higher human activity. *Mirounga leonine* is widely distributed in Southern hemisphere, with specimens found in the Antarctic Peninsula and continent, including Southern Argentina. *Leptonychotes weddellii* have a more circumpolar distribution, making foraging trips up north with expanding ice pack during the winter, and with some wandering as far as New Zealand, South Australia and South America. Finally, *Arctocephalus gazella* usually wander in non-breeding season to Weddell Sea, Argentina coast, and some groups have been reported in Juan Fernandez Island (Chile)^52,53^. Surveillance studies are needed to know the evolution of those EPEC/ExPEC clonal groups within the normal pinniped microbiota as well as their possible transmission to other animals within the Antarctic niches.

## Material and Methods

### *Escherichia* spp. collection

A total of 193 faecal samples from three Antarctic pinniped populations were collected during the month of February of years 2007, 2010 and 2011 in six locations with different human activity, from low to highly visited, along the west coast of the Antarctic Peninsula: Deception Island, King George Island, Livingston Island (Hannah Point and Byers Peninsula), Ronge Island and Avian Island in a latitudinal gradient covering 5° of latitude (ranging from 62°15′S; 58°37′W-67°46′S; 68°43′W), distances greater than 600 km and differences in mean annual temperatures of up to 2 °C. The populations sampled included individuals with several years of age (adults or subadults from both sexes) of Weddell seals (*Leptonychotes weddellii*), Southern elephant seals (*Mirounga leonina*) and Antarctic fur seals (*Arctocephalus gazella*). Samples consisted of fresh faecal samples obtained directly from the ground close to animals or swab samples from the rectum of animals randomly selected, captured and physically restrained. All captured animals were tagged to ensure that no animal was sampled more than once. Faecal samples were preserved in FBP medium^54^ with 0.5% active charcoal (Sigma Ltd.) and frozen at −20 °C until analysis in the lab. For the analysis, each sample was thawed and placed in 5 ml of buffered peptone water broth (Oxoid). After incubation (37 °C for 18 h), an aliquot of 100 µl was plated onto MacConkey agar (Oxoid) and plates incubated (37 °C for 24 h). One suspected *E*. *coli* colony per sample was randomly selected and identified by classical biochemical methods (gram-staining, triple sugar iron, citrate, urea, indol). Confirmed colonies were stored at −80 °C until their subsequent characterization.

Finally, a collection of 161 *Escherichia* spp. isolates from 160 pinnipeds (80 Antarctic fur seals, 61 Sourthern elephant seals and 19 Weddell seals; 94 faeces and 66 rectal swabs) was analysed, including two different colonies from a rectal swab of a Sourthern elephant seal (Supplementary Table S1). Human and other animal isolates from the own LREC-USC collections were used for comparative purposes.

### Typing, subtyping and phylogenetic grouping of the isolates

The isolates were characterized with regard to O:H antigens with all available O (O1–O181) and H (H1–H56) antisera^55^. Isolates that did not react with any antisera were designated as ONT or HNT (NT = nontypeable). Nonmotile isolates or cross-reactions were further tested by PCR using specific primers (Supplementary Table S4). The phylogenetic groups were determined by the quadruplex method of Clermont^56^ and those isolates that did not conform to any *E*. *coli* phylogroup were further investigated for the cryptic *Escherichia* lineages^57^ and *E*. *albertii*^58^.

All isolates were screened by PCR for the presence of the VF *eae*, *vt*_1_, *vt*_2_, *ipaH*, pcDV432, *eltA*, *est* or *stb* associated with the main intestinal pathotypes (enteropathogenic, verotoxigenic, enteroinvasive, enteroaggregative and enterotoxigenic) of *E*. *coli*. Likewise, specific extraintestinal VF were analyzed: *fimH*, *fimAv*_*MT78*_, *papC*, (positive results tested for *papG I*, *papG II* and *papG III*), *sfa/focDE* (positive results tested for *sfaS* and *focG*), *afa/draBC*, *cnf1*, *cdtB*, *sat*, *hlyA*, *iucD*, *iroN*, *kpsM II* (establishing *neuC*-K1, K2 and K5 variants), *kpsM III*, *cvaC*, *iss*, *traT*, *ibeA*, *malX*, *usp* and *tsh*. *E*. *coli* isolates were designated presumptively as extraintestinal pathogenic *E*. *coli* (ExPEC status)^13^ if positive for two or more of five markers: *papC*, *sfa/focDE*, *afa/draBC*, *kpsM II*, and *iutA* (Supplementary Table S5).

Isolates identified as ExPEC and/or *eae* carriers were further typed for their *fimH* alleles (type 1 fimbrial adhesion) with the amplification and sequencing of a 489-nucleotide internal fragment as described elsewhere^36^, which were compared with those available in the Genomic Epidemiology repository https://bitbucket.org/genomicepidemiology/fimtyper_db/downloads/. The *eae* variant was established by amplification and sequencing of the variable region of the gene (Supplementary Table S5). Furthermore, ExPEC and *eae*-positive isolates were analyzed for their STs following the MLST schemes of Achtman^14^ (http://enterobase.warwick.ac.uk/species/ecoli/allele_st_search) and additionally, for those ST131, of the Pasteur Institute^15^ (http://bigsdb.pasteur.fr/ecoli/ecoli.html). To analyze the phylogenetic relationships among the isolates, a Neighbor-Joining tree was constructed by MEGA6^59^ based on the concatenated sequences of the seven housekeeping genes for each isolate. Also, the virotype of the ST131 isolates was established based on the presence or absence of 13 specific VF according to the scheme described by Dahbi *et al*.^16^.

The *XbaI*-PFGE profiles of the ExPEC isolates were determined according to PulseNet protocol (http://www.pulsenetinternational.org/), imported into BioNumerics (Applied Maths, St-Martens-Latern Belgium) and clustered using the UPGMA algorithm and applying Dice coefficient and 1% tolerance.

### Antimicrobial susceptibility testing and resistance genes

Antimicrobial susceptibility was tested using the commercial broth microdilution minimum inhibitory concentration (MIC) panels for gram negatives in a MicroScan WalkAway automated system (Siemens Healthcare Diagnostics, CA, USA) according to the manufacturer’s instructions. The antibiotics tested included ampicillin, cefazolin, cefalotin, cefuroxime, cefotaxime, ceftazidime, cefepime, amoxicillin-clavulanic acid, piperacillin-tazobactam, cefoxitin, imipenem, gentamicin, tobramycin, nalidixic acid, norfloxacin, ciprofloxacin, trimethoprim-sulfamethoxazole, nitrofurantoin and fosfomycin. The MICs were interpreted according to standard break points^60^. Additionally, detection of TEM, SHV and CTX-M genes was performed by PCR using specific primers (Supplementary Table S6).

### Genome sequencing, assembly and analysis

DNA from ST131 Cplx isolates was extracted with the QIAamp 96 DNA Qiacube HT kit (Qiagen, Hilden, Germany) and libraries were prepared using the Nextera XT kit (Illumina). Pooled libraries were denatured following the Illumina protocol and 600 µl (approx. 20 pM) onto a MisSeq V2 -500 cycle cartridge (Illumina) and sequenced on a MiSeq to produces fastq files. Raw reads were uploaded and automatically assembled in Enterobase using SPAdes Genome Assembler v 3.5. with a contig threshold of minimum 200 nucleotides. Subsequently, the *de novo* assembled contigs were MLST (7 gene Achtman ST scheme, whole genome MLST and core genome MLST) and serotyped *in silico* using Enterobase typing tools (https://bitbucket.org/enterobase/enterobase-web/wiki/Home). A minimum spanning tree was constructed in Enterobase using the MSTree V2 algorithm^61^ and the cgMLST v1 scheme. This scheme consists of 2,513 genes that were present in over 98% of 3,457 genomes, which represented most of the diversity in Enterobase at the time (May 2016) https://bitbucket.org/enterobase/enterobase-web/wiki/Escherichia%20Statistics. Antimicrobial resistances, plasmid detection and typing were *in silico* determined by using ResFinder V2.1^62^ and PlasmidFinder 1.3./pMLST 1.4.^63^, respectively.

### Statistical analysis

Differences in the prevalence between groups were compared by a two-tailed Fisher’s exact test. *P* values < 0.05 were considered statistically significant.

### Ethics statement

Permissions for captures and sampling of the Antartic pinnipeds were granted by the Spanish Polar Committee CPE- EIA-2006-2 and CPE-EIA-2008-9, complying with the Antarctic Treaty System.

### Data availability

The whole genome sequenced samples are part of BioProject PRJEB19190 and correspond to BioSample IDs SAMEA92068168, SAMEA92071168, SAMEA92071918, SAMEA92086168, SAMEA92089168, SAMEA92092918, SAMEA92095168, SAMEA92096668, SAMEA92097418, SAMEA92099668.

## Electronic supplementary material

Supplementary Information
