# Supplementary material for: Impact of human-associated Escherichia coli clonal groups in Antarctic pinnipeds: presence of ST73, ST95, ST141 and ST131
Source: Sci Rep. 2018 Mar 16;8:4678. doi: 10.1038/s41598-018-22943-0 (PMC5856829; doi:10.1038/s41598-018-22943-0)
Supplement: Supplementary file 1 — Supplementary Information [file 41598_2018_22943_MOESM1_ESM.pdf]

Supplementary Information for

Impact of human-associated *Escherichia coli* clonal groups in Antarctic pinnipeds: presence of ST73, ST95, ST141 and ST131

Azucena Mora[1,*], Francisco Javier García-Peña[2], María Pilar Alonso[3], Susana Pedraza-Diaz[4,#], Luis Miguel Ortega-Mora[4], Daniel Garcia-Parraga[5], Cecilia López[1], Susana Viso[1], Ghizlane Dahbi[1], Juan Marzoa[1], Martin J. Sergeant[6], Vanesa García[1], Jorge Blanco[1]

[1]Laboratorio de Referencia de Escherichia coli (LREC), Departamento de Microbioloxía e Parasitoloxía, Facultade de Veterinaria, Universidade de Santiago de Compostela (USC), Lugo, Spain; [2]Laboratorio Central de Veterinaria de Algete, Ministerio de Agricultura y Pesca, Alimentación y Medio Ambiente, Ctra Madrid-Algete km 8, Madrid, Spain; [3]Servicio de Microbiología, Hospital Universitario Lucus Augusti (HULA), Lugo, Spain; [4]SALUVET, Animal Health Department, Faculty of Veterinary Sciences, Complutense University of Madrid, Madrid, Spain; [5]L'Oceanogràfic, Ciudad de las Artes y las Ciencias, Junta de Murs i Valls, Valencia, Spain; [6]Microbiology and Infection Unit, Warwick Medical School, University of Warwick, Coventry, United Kingdom

[#]Current affiliation: Environmental Toxicology, National Center for Environmental Health (CNSA), Institute of Health Carlos III, Madrid, Spain;

Corresponding author. A. Mora. Laboratorio de Referencia de *Escherichia coli* (LREC), Departamento de Microbioloxía e Parasitoloxía, Facultade de Veterinaria, Universidade de Santiago de Compostela (USC), 27002 Lugo, Spain. E-mail: azucena.mora@usc.es

Supplementary Tables:

Table S1. ***Escherichia* spp. collection (N= 158 *E. coli* and three *E. albertii* isolates) obtained from 160 Antarctic pinnipeds, ordered by ExPEC status and phylogroup**

Isolate code	Origin of isolation	Sample type	Location type[a]	Geographic location	Serotype[b]	FG[c]	ExPEC[d]	Virulence gene profile (virotype)[e]	Antibiotic Resistances[f]
EC-3	Southern elephant seal	Faeces	Rarely visited	Byers Peninsula	O145:H25	A	-	*fimH fimAvMT78 iutA iucD traT eae*-ε1	
EC-95	Southern elephant seal	Faeces	Moderate activity	King George Island	O145:H25	A	-	*fimH fimAvMT78 iutA iucD traT eae*-ε1	
EC-78	Southern elephant seal	Faeces	Rarely visited	Avian Island	O145:H25	A	-	*fimH iutA iucD traT eae*-ε1	
EC-61	Southern elephant seal	Faeces	Rarely visited	Avian Island	O8:HNM	A	-	*fimH fimAvMT78 iutA iucD traT eae*-θ1	
EC-64	Antarctic fur seal	Faeces	Rarely visited	Avian Island	O8:HNM	A	-	*fimH fimAvMT78 iutA iucD traT eae*-θ1	
EC-147	Antarctic fur seal	Faeces	Highly visited	Deception Island	O49:H10	A	-	*fimH iutA iucD traT eae*-k *bfp*	
EC-167	Antarctic fur seal	Rectal swab	Highly visited	Deception Island	O21:H9	B1	-	*fimH traT*	
EC-103	Weddell seal	Faeces	Highly visited	Deception Island	O15:H21	B1	-	*fimH*	
EC-105	Antarctic fur seal	Rectal swab	Highly visited	Deception Island	O15:H21	B1	-	*fimH*	
EC-118	Antarctic fur seal	Faeces	Highly visited	Deception Island	O15:H21	B1	-	*fimH*	
EC-150	Antarctic fur seal	Faeces	Highly visited	Deception Island	O15:H21	B1	-	*fimH*	
EC-153	Weddell seal	Faeces	Highly visited	Deception Island	O15:H21	B1	-	*fimH*	
EC-154	Antarctic fur seal	Faeces	Highly visited	Deception Island	O15:H21	B1	-	*fimH*	
EC-136	Antarctic fur seal	Rectal swab	Highly visited	Deception Island	O55,91:H11	B1	-	*fimH cdt3 traT malX eae*-κ	
EC-137	Weddell seal	Rectal swab	Highly visited	Deception Island	O55,91:H11	B1	-	*fimH cdt3 traT malX eae*-κ	
EC-138	Weddell seal	Rectal swab	Highly visited	Deception Island	O55,91:H11	B1	-	*fimH cdt3 traT malX eae*-κ	
EC-113	Weddell seal	Rectal swab	Highly visited	Deception Island	ONT:H16	B1	-	*fimH*	
EC-75	Southern elephant seal	Faeces	Rarely visited	Avian Island	ONT:H16	B1	-	*fimH*	
EC-180	Antarctic fur seal	Rectal swab	Highly visited	Deception Island	ONT:H2	B1	-	*fimH*	
EC-129	Antarctic fur seal	Rectal swab	Highly visited	Deception Island	ONT:H21	B1	-	*fimH traT*	
EC-51	Weddell seal	Rectal swab	Rarely visited	Avian Island	ONT:H21	B1	-	*fimH traT*	CEF*
EC-127	Antarctic fur seal	Faeces	Highly visited	Deception Island	ONT:H21	B1	-	*fimH*	
EC-157	Weddell seal	Rectal swab	Highly visited	Deception Island	ONT:H21	B1	-	*fimH*	
EC-159	Weddell seal	Rectal swab	Highly visited	Deception Island	ONT:H21	B1	-	*fimH*	
EC-161	Antarctic fur seal	Rectal swab	Highly visited	Deception Island	ONT:H21	B1	-	*fimH*	
EC-170	Weddell seal	Rectal swab	Highly visited	Deception Island	ONT:H21	B1	-	*fimH*	
EC-171	Weddell seal	Rectal swab	Highly visited	Deception Island	ONT:H21	B1	-	*fimH*	

Isolate code	Origin of isolation	Sample type	Location type[a]	Geographic location	Serotype[b]	FG[c]	ExPEC[d]	Virulence gene profile (virotype)[e]	Antibiotic Resistances[f]
EC-172a	Southern elephant seal	Rectal swab	Highly visited	Deception Island	ONT:H21	B1	-	*fimH*	
EC-92	Southern elephant seal	Faeces	Moderate activity	King George Island	ONT:H21	B1	-	*fimH traT malX*	
EC-90	Southern elephant seal	Faeces	Moderate activity	King George Island	O33:HNM	B2	-	*fimH fimAvMT78 cdt1 iutA iucD traT malX usp eae-β2*	
EC-91	Southern elephant seal	Faeces	Moderate activity	King George Island	O33:HNM	B2	-	*fimH fimAvMT78 cdt1 iutA iucD traT malX usp eae-β2*	
EC-181	Antarctic fur seal	Rectal swab	Highly visited	Deception Island	O4:H5	B2	-	*fimH kpsM III malX usp tsh*	
EC-38	Weddell seal	Rectal swab	Rarely visited	Rongé Island	O51:HNM	B2	-	*fimH cdt3 iutA iucD eae*-k	
EC-52	Weddell seal	Rectal swab	Rarely visited	Avian Island	O88:HNM	B2	-	*fimH438 cdt3 iutA iucD malX eae*-k	
EC-104	Weddell seal	Rectal swab	Highly visited	Deception Island	O120:H1	B2	-	*fimH malX usp*	
EC-128	Antarctic fur seal	Faeces	Highly visited	Deception Island	O120:H1	B2	-	*fimH malX usp*	
EC-162	Antarctic fur seal	Rectal swab	Highly visited	Deception Island	O120:H1	B2	-	*fimH malX usp*	
EC-163	Antarctic fur seal	Rectal swab	Highly visited	Deception Island	O120:H1	B2	-	*fimH malX usp*	
EC-166	Antarctic fur seal	Rectal swab	Highly visited	Deception Island	O120:H1	B2	-	*fimH malX usp*	
EC-168	Antarctic fur seal	Rectal swab	Highly visited	Deception Island	O120:H1	B2	-	*fimH malX usp*	
EC-191	Antarctic fur seal	Faeces	Highly visited	Deception Island	O120:H1	B2	-	*fimH malX usp*	
EC-151	Antarctic fur seal	Faeces	Highly visited	Deception Island	O120:H10	B2	-	*fimH traT malX usp*	
EC-133	Antarctic fur seal	Rectal swab	Highly visited	Deception Island	O149:H45	B2	-	*fimH cdt4 traT malX*	
EC-152	Antarctic fur seal	Faeces	Highly visited	Deception Island	O16:H5	B2	-	*fimH41 kpsM II-K5 malX usp* (NT)	
EC-190	Antarctic fur seal	Faeces	Highly visited	Deception Island	O39:H4	B2	-	*fimH21 fimAvMT78 kpsM II-K5 ibeA malX usp*	
EC-148	Antarctic fur seal	Faeces	Highly visited	Deception Island	O51:H49	B2	-	*fimH iutA iucD traT tsh eae*-α1	
EC-134	Antarctic fur seal	Rectal swab	Highly visited	Deception Island	O51:H49	B2	-	*fimH iutA iucD traT eae*-α1	
EC-156	Weddell seal	Rectal swab	Highly visited	Deception Island	O51:H49	B2	-	*fimH iutA iucD traT eae*-α1	
EC-165	Antarctic fur seal	Rectal swab	Highly visited	Deception Island	O51:H49	B2	-	*fimH iutA iucD traT eae*-α1	
EC-67	Southern elephant seal	Faeces	Rarely visited	Avian Island	O51:H49	B2	-	*fimH iutA iucD traT eae*-α1	
EC-82	Southern elephant seal	Faeces	Rarely visited	Avian Island	O51:H49	B2	-	*fimH iutA iucD traT eae*-α1	
EC-70	Southern elephant seal	Faeces	Rarely visited	Avian Island	O6:H1	B2	-	*fimH kpsM II-K5 malX usp tsh*	
EC-12	Southern elephant seal	Faeces	Rarely visited	Byers Peninsula	O78:HNT	B2	-	*fimH malX tsh*	CEF*
EC-87	Antarctic fur seal	Faeces	Moderate activity	King George Island	O78:HNT	B2	-	*fimH malX*	
EC-93	Southern elephant seal	Faeces	Moderate activity	King George Island	O78:HNT	B2	-	*fimH malX*	CEF*
EC-178a	Antarctic fur seal	Rectal swab	Highly visited	Deception Island	O80:H11	B2	-	*fimH iutA iucD traT ibeA usp*	
EC-44	Antarctic fur seal	Rectal swab	Rarely visited	Avian Island	O88:H1	B2	-	*fimH iroN malX usp*	
EC-13	Southern elephant seal	Faeces	Rarely visited	Byers Peninsula	O88:H10	B2	-	*fimH fimAvMT78 focG cdt4 cnf1 hlyA iroN ibeA malX*	

Isolate code	Origin of isolation	Sample type	Location type[a]	Geographic location	Serotype[b]	FG[c]	ExPEC[d]	Virulence gene profile (virotype)[e]	Antibiotic Resistances[f]
EC-14	Southern elephant seal	Faeces	Rarely visited	Byers Peninsula	O88:H10	B2	-	*fimH fimAvMT78 focG cdt4 cnf1 hlyA iroN ibeA malX*	CEF*
EC-97	Southern elephant seal	Faeces	Moderate activity	King George Island	ONT:H4	B2	-	*fimH kpsMII-K2 traT malX usp*	
EC-1	Southern elephant seal	Faeces	Rarely visited	Byers Peninsula	O141:H18	D	-	*fimH kpsM II-K5*	
EC-22	Southern elephant seal	Faeces	Highly visited	Hannah Point	O141:H18	D	-	*fimH kpsM II-K5*	
EC-101	Southern elephant seal	Faeces	Moderate activity	King George Island	O141:H45	D	-	*fimH kpsM II-K5*	NAL
EC-102	Southern elephant seal	Faeces	Moderate activity	King George Island	O141:H45	D	-	*fimH kpsM II-K5*	
EC-164	Antarctic fur seal	Rectal swab	Highly visited	Deception Island	O44:H18	D	-	*fimH kpsM II-K2 traT tsh*	
EC-117	Antarctic fur seal	Faeces	Highly visited	Deception Island	O44:H18	D	-	*fimH kpsM II-K2 tsh*	
EC-187	Antarctic fur seal	Faeces	Highly visited	Deception Island	O44:H18	D	-	*fimH kpsM II-K2 tsh*	
EC-96	Southern elephant seal	Faeces	Moderate activity	King George Island	O73:H18	D	-	*fimH traT*	
EC-68	Southern elephant seal	Faeces	Rarely visited	Avian Island	O73:H45	D	-	*fimH traT*	
EC-11	Southern elephant seal	Faeces	Rarely visited	Byers Peninsula	O73:H45	D	-	*fimH*	
EC-2	Southern elephant seal	Faeces	Rarely visited	Byers Peninsula	O73:H45	D	-	*fimH*	CEF*
EC-59	Antarctic fur seal	Faeces	Rarely visited	Avian Island	O73:HNM	D	-	*fimH fimAvMT78 traT*	
EC-16	Southern elephant seal	Faeces	Rarely visited	Byers Peninsula	O73:HNM	D	-	*fimH traT*	
EC-60	Antarctic fur seal	Faeces	Rarely visited	Avian Island	O73:HNM	D	-	*fimH traT*	
EC-35	Southern elephant seal	Faeces	Highly visited	Hannah Point	O77:H45	D	-	*fimH iutA iucD*	
EC-36	Southern elephant seal	Faeces	Highly visited	Hannah Point	O77:H45	D	-	*fimH iutA iucD*	
EC-4	Southern elephant seal	Faeces	Rarely visited	Byers Peninsula	O77:H45	D	-	*fimH iutA iucD*	
EC-6	Southern elephant seal	Faeces	Rarely visited	Byers Peninsula	O77:H45	D	-	*fimH iutA iucD*	
EC-74	Southern elephant seal	Faeces	Rarely visited	Avian Island	O77:H45	D	-	*fimH traT*	
EC-80	Southern elephant seal	Faeces	Rarely visited	Avian Island	O77:H45	D	-	*fimH iutA iucD traT*	
EC-7	Southern elephant seal	Faeces	Rarely visited	Byers Peninsula	O77:HNM	D	-	*fimH traT*	AMP*
EC-112	Antarctic fur seal	Rectal swab	Highly visited	Deception Island	O15:H1	D	-	*fimH fimAvMT78 kpsM II-K5 tsh*	
EC-149	Antarctic fur seal	Faeces	Highly visited	Deception Island	O15:H1	D	-	*fimH fimAvMT78 kpsM II-K5 traT*	
EC-15	Southern elephant seal	Faeces	Rarely visited	Byers Peninsula	O15:H1	D	-	*fimH fimAvMT78 kpsM II-K5*	
EC-122	Antarctic fur seal	Faeces	Highly visited	Deception Island	O15:H45	D	-	*fimH fimAvMT78 kpsM II-K5 traT malX tsh*	
EC-28	Southern elephant seal	Faeces	Highly visited	Hannah Point	O73:H34	E	-	*fimH kpsM II-K5*	
EC-192	Antarctic fur seal	Faeces	Highly visited	Deception Island	O73:H34	E	-	*fimH kpsM II-K5*	
EC-179a	Antarctic fur seal	Rectal swab	Highly visited	Deception Island	O73:HNM	E	-	*fimH kpsM II-K5*	
EC-193	Antarctic fur seal	Faeces	Highly visited	Deception Island	O73:HNM	E	-	*fimH kpsM II-K5*	

Isolate code	Origin of isolation	Sample type	Location type[a]	Geographic location	Serotype[b]	FG[c]	ExPEC[d]	Virulence gene profile (virotype)[e]	Antibiotic Resistances[f]
EC-120	Antarctic fur seal	Faeces	Highly visited	Deception Island	O77:H34	E	-	*fimH kpsM II-K5*	
EC-121	Antarctic fur seal	Faeces	Highly visited	Deception Island	O77:H34	E	-	*fimH kpsM II-K5*	
EC-85	Southern elephant seal	Faeces	Moderate activity	King George Island	O9:H20	F	-	*fimH fimAvMT78 malX*	
EC-86	Southern elephant seal	Faeces	Moderate activity	King George Island	O9:H20	F	-	*fimH fimAvMT78 malX*	CEF*
EC-132	Antarctic fur seal	Rectal swab	Highly visited	Deception Island	O15:HNT	F	-	*fimH iutA iucD traT malX*	
EC-143	Antarctic fur seal	Rectal swab	Highly visited	Deception Island	O15:HNT	F	-	*fimH iutA iucD traT malX*	
EC-98	Antarctic fur seal	Faeces	Moderate activity	King George Island	O48,87:H25	F	-	*fimH kpsM II-K2 trat malX*	
EC-76	Southern elephant seal	Faeces	Rarely visited	Avian Island	O84:HNM	*	-	*fimH fimAvMT78 cdt3 iutA iucD traT eae*-ο	
EC-25	Southern elephant seal	Faeces	Highly visited	Hannah Point	ONT:HNM	*	-	*fimH cdt3 iutA iucD traT eae*-ο	CEF
EC-172b	Southern elephant seal	Rectal swab	Highly visited	Deception Island	O128:HNM	*	-	*fimH cdt3 iutA iucD traT ibeA eae*-κ	
EC-88	Antarctic fur seal	Faeces	Moderate activity	King George Island	O88:H25	B1	+	*fimH focG iutA iucD iroN traT eae*-β1	NAL
EC-48	Southern elephant seal	Rectal swab	Rarely visited	Avian Island	O46:H31	B2	+	*fimH cdt4 iutA iucD kpsM II-K1 traT ibeA malX usp*	CEF*
EC-26	Southern elephant seal	Faeces	Highly visited	Hannah Point	O4:H5	B2	+	*fimH papG III sfaS cnf1 hlyA iroN traT malX usp*	
EC-50	Antarctic fur seal	Rectal swab	Rarely visited	Avian Island	O4:H5	B2	+	*fimH papG III sfaS cnf1 hlyA iroN traT malX usp*	
EC-186	Antarctic fur seal	Faeces	Highly visited	Deception Island	O4:H5	B2	+	*fimH papG III sfaS cnf1 hlyA iroN malX usp*	
EC-184	Antarctic fur seal	Rectal swab	Highly visited	Deception Island	O88:H5	B2	+	*fimH fimAvMT78 papG I sfaS traT ibeA malX usp*	
EC-54	Antarctic fur seal	Rectal swab	Rarely visited	Avian Island	ONT:H6	B2	+	*fimH fimAvMT78 sfaS cnf1 hlyA iutA iucD iroN kpsM II-K1 traT ibeA malX usp*	
EC-43	Antarctic fur seal	Rectal swab	Rarely visited	Avian Island	O120:HNM	B2	+	*fimH fimAvMT78 papG I sfaS cnf1 hlyA kpsM II-K1 ibeA malX usp*	
EC-100	Southern elephant seal	Faeces	Moderate activity	King George Island	O129:H4	B2	+	*fimH iutA iucD kpsM II-K2 malX usp*	CEF*
EC-41	Antarctic fur seal	Rectal swab	Rarely visited	Avian Island	O129:H4	B2	+	*fimH iutA iucD iroN kpsM II-K2 traT malX usp*	
EC-46	Antarctic fur seal	Rectal swab	Rarely visited	Avian Island	O2:H1	B2	+	*fimH papG III sfaS cnf1 hlyA iutA iucD iroN kpsM II-K5 traT malX usp*	CEF*
EC-19	Southern elephant seal	Faeces	Highly visited	Hannah Point	O2:H6	B2	+	*fimH sfaS iutA iucD iroN kpsM II-K1 traT malX usp*	CEF*
EC-111	Antarctic fur seal	Rectal swab	Highly visited	Deception Island	O2:H6	B2	+	*fimH sfaS cdt1 iroN kpsM II-K1 ibeA malX usp*	
EC-99	Southern elephant seal	Faeces	Moderate activity	King George Island	O2:H7	B2	+	*fimH papG III cnf1 hlyA kpsM II-K1 traT malX usp*	
EC-23	Southern elephant seal	Faeces	Highly visited	Hannah Point	O25b:H4	B2	+	*fimH papG III cdt4 iutA iucD iroN kpsM II-K5 cvaC iss traT ibeA malX usp* (D2)	AMP CEF*
EC-24	Southern elephant seal	Faeces	Highly visited	Hannah Point	O25b:H4	B2	+	*fimH papG III cdt4 iutA iucD iroN kpsM II-K5 cvaC iss traT ibeA malX usp* (D2)	AMP CEF
EC-108	Antarctic fur seal	Rectal swab	Highly visited	Deception Island	O25b:H4	B2	+	*fimH papG II iutA iucD iroN kpsM II-K1 cvaC iss traT malX usp*	
EC-77	Southern elephant seal	Faeces	Rarely visited	Avian Island	O25b:H4	B2	+	*fimH sfaS cdt4 iutA iucD kpsM II-K5 traT ibeA malX usp tsh* (D1)	
EC-115	Antarctic fur seal	Rectal swab	Highly visited	Deception Island	O25b:H4	B2	+	*fimH cdt4 iutA iucD kpsM II-K2 traT ibeA malX usp tsh* (D-nt)	CEF*
EC-114	Weddell seal	Rectal swab	Highly visited	Deception Island	O25b:H4	B2	+	*fimH cdt4 iutA iucD kpsM II-K2 traT ibeA malX usp* (D-nt)	
EC-160	Antarctic fur seal	Rectal swab	Highly visited	Deception Island	O25b:H4	B2	+	*fimH cdt4 iutA iucD kpsM II-K2 traT ibeA malX usp* (D-nt)	

Isolate code	Origin of isolation	Sample type	Location type[a]	Geographic location	Serotype[b]	FG[c]	ExPEC[d]	Virulence gene profile (virotype)[e]	Antibiotic Resistances[f]
EC-182	Antarctic fur seal	Rectal swab	Highly visited	Deception Island	O39:H5	B2	+	*fimH iutA iucD kpsM II-K5 ibeA malX usp*	
EC-131	Antarctic fur seal	Rectal swab	Highly visited	Deception Island	O6:H1	B2	+	*fimH papG III sfaS cnf1 cdt4 hlyA iroN kpsM II-K5 traT malX usp*	
EC-144	Antarctic fur seal	Faeces	Highly visited	Deception Island	O6:H1	B2	+	*fimH papG III sfaS cnf1 cdt4 hlyA iroN kpsM II-K5 traT malX usp*	
EC-32	Southern elephant seal	Faeces	Highly visited	Hannah Point	O6:H1	B2	+	*fimH focG iroN kpsM II-K5 traT malX usp*	
EC-79	Southern elephant seal	Faeces	Rarely visited	Avian Island	O6:H1	B2	+	*fimH fimAvMT78 focG iroN kpsM II-K5 malX usp*	
EC-106	Antarctic fur seal	Rectal swab	Highly visited	Deception Island	O6:H1	B2	+	*fimH focG iroN kpsM II-K5 malX usp*	
EC-107	Antarctic fur seal	Rectal swab	Highly visited	Deception Island	O6:H1	B2	+	*fimH focG iroN kpsM II-K5 malX usp*	
EC-135	Antarctic fur seal	Rectal swab	Highly visited	Deception Island	O6:H1	B2	+	*fimH focG iroN kpsM II-K5 malX usp*	
EC-155	Weddell seal	Rectal swab	Highly visited	Deception Island	O6:H1	B2	+	*fimH focG iroN kpsM II-K5 malX usp*	
EC-21	Southern elephant seal	Faeces	Highly visited	Hannah Point	O6:H1	B2	+	*fimH focG iroN kpsM II-K5 malX usp*	
EC-29	Southern elephant seal	Faeces	Highly visited	Hannah Point	O6:H1	B2	+	*fimH focG iroN kpsM II-K5 malX usp*	
EC-30	Southern elephant seal	Faeces	Highly visited	Hannah Point	O6:H1	B2	+	*fimH focG iroN kpsM II-K5 malX usp*	
EC-31	Southern elephant seal	Faeces	Highly visited	Hannah Point	O6:H1	B2	+	*fimH focG iroN kpsM II-K5 malX usp*	
EC-94	Southern elephant seal	Faeces	Moderate activity	King George Island	O6:H1	B2	+	*fimH focG iroN kpsM II-K5 malX usp*	
EC-173	Antarctic fur seal	Rectal swab	Highly visited	Deception Island	O6:H21	B2	+	*fimH papG III sfaS cnf1 hlyA iroN kpsM II-K5 traT malX usp*	
EC-146	Antarctic fur seal	Faeces	Highly visited	Deception Island	O6:H7	B2	+	*fimH papG III sfaS cnf1 hlyA iroN kpsM II-K5 traT ibeA malX usp*	
EC-176	Antarctic fur seal	Rectal swab	Highly visited	Deception Island	O6:HNM	B2	+	*fimH papG III sfaS cnf1 hlyA iroN kpsM II-K5 traT malX usp*	
EC-116	Weddell seal	Rectal swab	Highly visited	Deception Island	O77:H45	B2	+	*fimH focG iutA iucD iroN kpsM II-K5 malX usp*	
EC-34	Southern elephant seal	Faeces	Highly visited	Hannah Point	O78:H7	B2	+	*fimH sfaS iutA iucD iroN traT malX*	
EC-69	Southern elephant seal	Faeces	Rarely visited	Avian Island	O78:HNM	B2	+	*fimH sfa/focDE iutA iucD iroN traTmalX*	
EC-18	Southern elephant seal	Faeces	Rarely visited	Byers Peninsula	O78:HNT	B2	+	*fimH sfa/focDE iutA iucD iroN malX tsh*	
EC-119	Antarctic fur seal	Faeces	Highly visited	Deception Island	ONT:H34	B2	+	*fimH iutA iucD kpsM II-K5 traT ibeA malX usp*	
EC-139	Antarctic fur seal	Rectal swab	Highly visited	Deception Island	ONT:H4	B2	+	*fimH sfaS iutA traT ibea malX usp*	
EC-140	Antarctic fur seal	Rectal swab	Highly visited	Deception Island	ONT:H4	B2	+	*fimH sfaS iutA traT ibea malX usp*	
EC-141	Antarctic fur seal	Rectal swab	Highly visited	Deception Island	ONT:H4	B2	+	*fimH sfaS iutA traT ibea malX usp*	
EC-109	Antarctic fur seal	Rectal swab	Highly visited	Deception Island	ONT:H5	B2	+	*fimH fimAvMT78 iutA iucD kpsM II-K5 ibeA malX usp*	
EC-10	Southern elephant seal	Faeces	Rarely visited	Byers Peninsula	ONT:H6	B2	+	*fimH fimAvMT78 sfaS cnf1 hlyA iutA iucD iroN kpsM II-K1 traT ibeA malX usp*	CEF*
EC-145	Antarctic fur seal	Faeces	Highly visited	Deception Island	ONT:H6	B2	+	*fimH fimAvMT78 sfaS cnf1 hlyA iutA iucD iroN kpsM II-K1 traT ibeA malX usp*	
EC-183	Antarctic fur seal	Rectal swab	Highly visited	Deception Island	ONT:H6	B2	+	*fimH fimAvMT78 sfaS cnf1 hlyA iutA iucD iroN kpsM II-K1 traT ibeA malX usp*	
EC-45	Antarctic fur seal	Rectal swab	Rarely visited	Avian Island	ONT:H6	B2	+	*fimH fimAvMT78 sfaS cnf1 hlyA iutA iucD iroN kpsM II-K1 traT ibeA malX usp*	
EC-47	Southern elephant seal	Rectal swab	Rarely visited	Avian Island	ONT:H6	B2	+	*fimH fimAvMT78 sfaS cnf1 hlyA iutA iucD iroN kpsM II-K1 traT ibeA malX usp*	

Isolate code	Origin of isolation	Sample type	Location type[a]	Geographic location	Serotype[b]	FG[c]	ExPEC[d]	Virulence gene profile (virotype)[e]	Antibiotic Resistances[f]
EC-8	Southern elephant seal	Faeces	Rarely visited	Byers Peninsula	ONT:H6	B2	+	*fimH fimAvMT78 sfaS cnf1 hlyA iutA iucD iroN kpsM II-K1 traT ibeA malX usp*	
EC-56	Antarctic fur seal	Rectal swab	Rarely visited	Avian Island	ONT:H6	B2	+	*fimH fimAvMT78 sfaS cnf1 hlyA iutA iucD iroN kpsM II-K1 traT ibeA malX*	CEF*
EC-83	Southern elephant seal	Faeces	Rarely visited	Avian Island	O44:H41	E	+	*fimH iutA iucD kpsM II-K5 traT*	
EC-158	Weddell seal	Rectal swab	Highly visited	Deception Island	O73:H34	E	+	*fimH iutA iucD kpsM II-K5 traT*	
EC-142	Antarctic fur seal	Rectal swab	Highly visited	Deception Island	O11:H6	F	+	*fimH iutA iucD iroN kpsM II-K5 cvaC iss traT ibeA malX usp*	
EC-123	Antarctic fur seal	Faeces	Highly visited	Deception Island	O11:H6	F	+	*fimH iutA iucD kpsM II-K5 traT malX*	
EC-124	Antarctic fur seal	Faeces	Highly visited	Deception Island	O11:H6	F	+	*fimH iutA iucD kpsM II-K5 traT malX*	
EC-125	Antarctic fur seal	Faeces	Highly visited	Deception Island	O11:H6	F	+	*fimH iutA iucD kpsM II-K5 traT malX*	
EC-130	Antarctic fur seal	Rectal swab	Highly visited	Deception Island	O11:H6	F	+	*fimH iutA iucD kpsM II-K5 traT malX*	
EC-126	Weddell seal	Faeces	Highly visited	Deception Island	O15:HNT	F	+	*fimH iutA iucD kpsM II-K5 traT malX*	CEF

[a] Human activity within the six locations sampled, from low to highly visited.

[b] O antigen: nontypeable isolates were designated as ONT; H antigen: HNM for nonmotile isolates and HNT for those which did not react with any antisera.

[c] Phylogroups designated by PCR according to Clermont *et al*. scheme[1]. * = *Escherichia albertii* isolates: these three isolates (EC-25, EC-76 and EC-172b) could not be classified with the quadriplex PCR which for EC-15 and EC-76 was: *arpA*-, *chuA*-, *yjaA*- and TspE4-, while for EC-172b was: *arpA*-, *chuA*-, *yjaA*- and TspE4+; additionally, the three showed the same band of 450-500 bp, corresponding to the described PCR product of *chuA* obtained with the chuA.2 and Acek.f primers. Finally, the three were classified as *E. albertii* by means of specific PCR[2,3].

[d] ExPEC +: strains presumptively regarded as extraintestinal pathogenic *E. coli* since they were positive for two or more of five markers, including *papAH* and/or *papC*, *sfa/focDE*, *afa/draBC*, *kpsM II* and *iutA*; ExPEC -: strains negative for those markers[4].

[e] Virotype was established for those isolates belonging to the ST131 Cplx, based on the presence or absence of 13 specific VF from those screened according to the scheme described by Dahbi *et al*.[5]; NT: not typeable.

[f] Antimicrobial susceptibility was tested using the commercial broth microdilution minimum inhibitory concentration (MIC); the MICs were interpreted according to standard break points[6]; AMP: ampicillin, CEF: cefalotin, NAL: nalidixic acid, *Intermediate values.

Table S2. VF and phylogroup prevalences of the pinniped collection (N=161 *Escherichia* spp.). ExPEC status *vs* non-ExPEC comparison of the 158 *E. coli* isolates

VF and Phylogroups	Weddell seal No. (%)	Southern elephant seal No. (%)	Antarctic fur seal No. (%)	[a]TOTAL (%) N = 161	[b]ExPEC status N= 62 (%)	[c]Non-ExPEC status N=96 (%)	[d]ExPEC vs non-ExPEC *P* values
fimH	19 (100.0)	62 (100.0)	80 (100.0)	161 (100.0)	62 (100.0)	96 (100.0)	1
fimAv$_{MT78}$	0	15 (24.2)	14 (17.5)	29 (18.0)	12 (19.4)	16 (16.7)	0.832
papC	0	4 (6.5)	11 (13.8)	15 (9.3)	**15 (24.2)**	0	**<0.001**
papG I	0	0	2 (2.5)	2 (1.2)	2 (3.2)	0	0.152
papG II	0	0	1 (1.3)	1 (0.6)	1 (1.6)	0	0.392
papG III	0	4 (6.5)	8 (10.0)	12 (7.5)	**12 (19.4)**	0	**<0.001**
sfa/focDE	2 (10.5)	18 (29.0)	23 (28.8)	43 (26.7)	**41 (66.1)**	2 (2.1)	**<0.001**
sfaS	0	7 (11.3)	19 (23.8)	26 (16.1)	**26 (41.9)**	0	**<0.001**
focG	2 (10.5)	9 (14.5)	4 (5.0)	15 (9.3)	**13 (21.0)**	2 (2.1)	**<0.001**
afa/draBC	0	0	0	0	0	0	1
cnf1	0	7 (11.3)	14 (17.5)	21 (13.0)	**19 (30.6)**	2 (2.1)	**<0.001**
cdtB	5 (26.3)	11 (17.7)	7 (8.8)	23 (14.3)	10 (16.1)	10 (10.4)	0.332
sat	0	0	0	0	0	0	1
hlyA	0	7 (11.3)	14 (17.5)	21 (13.0)	**19 (30.6)**	2 (2.1)	**<0.001**
iutA	7 (36.8)	29 (46.8)	30 (37.5)	66 (41.0)	**39 (62.9)**	24 (25.0)	**<0.001**
iucD	7 (36.8)	29 (46.8)	27 (33.8)	63 (39.1)	**36 (58.1)**	24 (25.0)	**<0.001**
iroN	2 (10.5)	19 (30.6)	22 (27.5)	43 (26.7)	**40 (64.5)**	3 (3.1)	**<0.001**
kpsM II	5 (26.3)	26 (41.9)	42 (52.5)	73 (45.3)	**51 (82.3)**	22 (22.9)	**<0.001**
kpsM II-K2	1 (5.3)	2 (3.2)	7 (8.8)	10 (6.2)	5 (8.1)	5 (5.2)	0.515
kpsM II-K5	4 (21.1)	18 (29.0)	27 (33.8)	49 (30.4)	**31 (50.0)**	18 (18.7)	**<0.001**
neuC	0	6 (9.7)	8 (10.0)	14 (8.7)	**14 (22.6)**	0	**<0.001**
kpsM III	0	0	1 (1.3)	1 (0.6)	0	1 (1.0)	1
cvaC	0	2 (3.2)	2 (2.5)	4 (2.5)	**4 (6.5)**	0	**0.022**
iss	0	2 (3.2)	2 (2.5)	4 (2.5)	**4 (6.5)**	0	**0.022**
traT	7 (36.8)	33 (53.2)	46 (57.5)	86 (53.4)	**44 (71.0)**	39 (40.6)	**<0.001**
ibeA	1 (5.3)	10 (16.1)	20 (25.0)	31 (19.3)	**26 (41.9)**	4 (4.2)	**<0.001**
malX	8 (42.1)	32 (51.6)	52 (65.0)	92 (57.1)	**58 (93.5)**	34 (35.4)	**<0.001**
usp	4 (21.1)	22 (35.5)	41 (51.3)	67 (41.6)	**49 (79.0)**	18 (18.7)	**<0.001**
tsh	0	4 (6.5)	8 (10.0)	12 (7.5)	3 (4.8)	9 (9.4)	0.368
eae	5 (26.3)	11 (17.7)	7 (8.8)	23 (14.3)	1 (1.6)	**19 (19.8)**	**<0.001**
stx$_1$	0	0	0	0	0	0	1
stx$_2$	0	0	0	0	0	0	1
ipaH	0	0	0	0	0	0	1
pcDV432	0	0	0	0	0	0	1
eltA	0	0	0	0	0	0	1
est	0	0	0	0	0	0	1
stb	0	0	0	0	0	0	1
phylogroup A	0	4 (6.5)	2 (2.5)	6 (3.7)	0	6 (6.2)	0.082
**phylogroup B1**	10 (52.6)	3 (4.8)	11 (13.8)	24 (14.9)	1 (1.6)	**23 (24.0)**	**<0.001**
**phylogroup B2**	7 (36.8)	31 (50.0)	46 (57.5)	84 (52.2)	**53 (85.5)**	31 (32.3)	**<0.001**
**phylogroup D**	0	17 (27.4)	8 (10.0)	25 (15.5)	0	**25 (26.0)**	**<0.001**
phylogroup E	1 (5.3)	2 (3.2)	5 (6.3)	8 (5.0)	2 (3.2)	6 (6.2)	0.482
phylogroup F	1 (5.3)	2 (3.2)	8 (10.0)	11 (6.8)	6 (9.7)	5 (5.2)	0.346
[e]*E. albertii*	0	3 (4.8)	0	3 (1.9)			

Trait prevalences: [a]within the total collection (N=161 *Escherichia* spp.); [b]within the 62 *E. coli* isolates positive for the ExPEC status; [c]within the 96 *E. coli* non-ExPEC isolates; [d]*P* value by two-tailed Fisher´s exact test; **in bold** those traits and values that shown significant differences; [e]the three *E. albertii* isolates carried 6-7 VF but did not satisfied the ExPEC status defined for *E. coli*.

Table S3. MLST according to the Achtman scheme and number of isolates

adk	*fumC*	*gyrB*	*icd*	*mdh*	*purA*	*recA*	ST	ST Cplx	No. isolates
36	24	9	13	17	11	25	ST73	73	14
76	115	10	119	30	37	136	ST1859	None	8
53	40	47	13	36	28	29	ST131	131	6
13	39	13	13	18	10	79	ST589	None	6
101	88	97	108	26	79	2	ST457	None	5
13	13	9	13	16	10	9	ST12	12	3
6	4	12	16	9	7	7	ST29	29	3
1	46	new	1	20	new	7	STnew1	None	3
13	166	19	13	17	25	new	STnew6	None	3
13	52	10	14	17	25	17	ST141	None	2
94	324	85	169	111	69	76	ST2087	None	2
13	21	13	22	17	14	15	ST28	28	2
13	52	10	119	17	37	25	ST547	None	2
13	147	93	13	17	28	30	ST640	None	2
38	39	30	13	17	25	28	ST681	None	2
37	38	19	37	17	11	26	ST95	95	2
1	46	new	new	new	34	7	STnew2	None	2
13	14	19	36	23	11	10	ST127	None	1
38	24	30	13	17	25	28	ST1879	None	1
6	7	5	1	8	18	2	ST206	206	1
9	23	81	18	11	8	6	ST328	278	1
13	38	84	13	17	64	26	ST363	None	1
36	24	9	13	5	11	25	ST4446	None	1
13	566	10	13	16	318	25	ST4448	None	1
18	567	20	6	5	5	4	ST4449	None	1
35	3	20	6	5	16	4	ST4607	None	1
13	4	19	36	20	68	10	ST4610	None	1
13	39	121	13	17	10	10	ST4611	None	1
13	43	9	37	17	37	25	ST567	None	1
13	43	13	14	30	94	92	ST625	None	1
53	40	47	13	36	462	29	ST6252	131	1
92	4	87	96	70	58	2	ST648	648	1
new	568	247	new	17	11	93	STnew3	None	1
new	568	247	192	17	new	new	STnew4	None	1
new	393	85	483	109	66	75	STnew5	None	1

Table S4. Primers used for O and H antigen typing

Target	Primers	Nucleotide sequence (5´- 3´)	Size (bp)	Reference
*fli*C$_{H2}$	H2-F<br>H2-R	AACGACGGCGAAACAATTAC<br>AGAACGCAACGAGTCAACCT	828	Alonso *et al.* [7]
*fli*C$_{H4}$	H4-F<br>H4-R	GCAGCGTATTCGTGAACTGA<br>GCTGGATAATCTGCGCTTTC	713	Mora *et al.* [8]
*fli*C$_{H7}$	H7-F<br>H7-R	GCGCTGTCGAGTTCTATCGAGC<br>CAACGGTGACTTTATCGCCATTCC	625	Gannon *et al.* [9]
*fli*C$_{H8}$	H8-F<br>H8-R	TAACAGCGCAAAAGACGATG<br>CCGAGAGTTTTCGCATCAAT	393	Mora *et al.* [10]
*fli*C$_{H9}$	H9-F<br>H9-R	ACGAAATCAAATCCCGTCTG<br>GCGGTATCGTTACCTGCATT	649	Mora *et al.* [10]
*fli*C$_{H10}$	H10-F<br>H10-R	AGCAAGTGGCAGTAGGTGCT<br>GCTGGATAATCTGCGCTTTC	624	Alonso *et al.* [7]
*fli*C$_{H11}$	H11-F<br>H11-R	ACTGTTAACGTAGATAGC<br>TCAATTTCTGCAGAATATAC	248	Durso *et al.* [11]
*fli*C$_{H18}$	H18-F1<br>H18-R1	TTCTGACCTGGACTCCATCC<br>CGTTAGCAAACGTTGAAGCA	827	LREC-USC (this study)
*fli*C$_{H21}$	H21-F<br>H21-R3	GGCGATTGCTAACCGTTTTA<br>CGTAAGTGAACCATCCGCAG	549-556	Mora *et al.* [10]
*fli*C$_{H25}$	H25-F<br>H25-R	ATGAAATTGACCGCGTATCC<br>TTGCGGGATAGATGTGATAGC	212	Alonso *et al.* [7]
*rfb*O25b	rfb. 1bis.f<br>rfbO25b.r	ATACCGACGACGCCGATCTG<br>TGCTATTCATTATGCGCAGC	300	Clermont *et al.* [12]
wzx-O16	wzx-F<br>wzx-R	GGTTTCAATCTCACAGCAACTCAG<br>GTTAGAGGGATAATAGCCAAGCGG	302	Li *et al.* [13]

Table S5. Targets and primers associated with diarreagenic and extraintestinal pathotypes of *E. coli*

Pathotype	Target	Primers	Nucleotide sequence (5´- 3´)	Size (bp)	Reference
VTEC	vt_1	VT1-F	TCGCTGAATGTCATTCGCTCTGC	539	Mora *et al.* [8]
		VT1-R	TCAGCAGTCATTACATAAGAAC		
VTEC	vt_2	VT2-F1	TTTCTTCGGTATCCTATTCCC	358	Mora *et al.* [8]
		VT2-F2	TGTCTTCAGCATCTTATGCAG		
		Vt2-R	CTGCTGTCCGTTGTCATGGAA		
VTEC EPEC	*eae*	EAE-V3F	CATTGATCAGGATTTTTCTGGT	510	Mora *et al.* [8]
		EAE-MBR	TCCAGAATAATATTGTTATTACG		
VTEC EPEC	*eae*[a]	EAE-R11	TCTTCGGAGGGTTTTTTATT	1125	Alonso *et al.* [7]
		EAE-FBN	CAGGTCGTCGTGTCTGCTAAAAC		
VTEC EPEC	*eae*[a]	EAE-R12	CCAGACGAATATATACATATTC	1181	Alonso *et al.* [7]
		EAE-FBN	CAGGTCGTCGTGTCTGCTAAAAC		
tEPEC	*bfpA*	EP1	AATGGTGCTTGCGCTTGCTGC	326	Gunzburg *et al.* [14]
		EP2	GCCGCTTTATCCAACCTGGTA		
EIEC	*ipaH*	EI1	GCTGGAAAAACTCAGTGCCT	424	Tornieporth *et al.* [15]
		EI2	CCAGTCCGTAAATTCATTCT		
EAEC	pCDV432	pCVD432/start	CTGGCGAAAGACTGTATCAT	630	Schmidt *et al.* [16]
		pCVD432/stop	CAATGTATAGAAATCCGCTGTT		
ETEC	*eltA*	LT-A-1	GGCGACAGATTATACCGTGC	696	Schultsz *et al.* [17]
		LT-A-2	CCGAATTCTGTTATATATGTC		
ETEC	*est*	STa-A	ATTTTTATTTCTGTATTGTCTTT	176	Penteado *et al.* [18]
		STa-B	GGATTACAACACAGTTCACAGCAGT		
ETEC	*stb*	STb-1	ATCGCATTTCTTCTTGCATC	172	Blanco et al. [19]
		STb-2	GGGCGCCAAAGCATGCTCC		
ExPEC	*fimH*	FimH f	TGCAGAACGGATAAGCCGTGG	508	Johnson & Stell [20]
		FimH r	GCAGTCACCTGCCCTCCGGTA		
ExPEC	$fimAv_{MT78}$	fimA201	TCTGGCTGATACTACACC	266	Marc & Dho-Moulin [21]
		fimA215	ACTTTAGGATGAGTACTG		
ExPEC	*papC*	pap1	GACGGCTGTACTGCAGGGTGTGGCG	328	Blanco *et al.* [19]
		pap2	ATATCCTTTCTGCAGGGATGCAATA		
ExPEC	*papG I*	pap-I F	TTAGCTGGATGGCACAATG	335	Mora *et al.* [22]
		pap-I R	TTGTCCATGTATCCCATTCAT		
ExPEC	*papG II*	pap-II F	GGGCATTGCTACGGTAACCTG	545	Mora *et al.* [22]
		pap-II R	CGCTATTAATAGACAGATCACC		
ExPEC	*papG III*	pap-III F	CGGCAACTTTAAGCTATGTG	720	Mora *et al.* [22]
		pap-III R	TGTACCATCTCATCGTTGTCTC		
ExPEC	*sfa/focDE*	sfa1	CTCCGGAGAACTGGGTGCATCTTAC	410	Le Bouguenec *et al.* [23]
		sfa2	CGGAGGAGTAATTACAAACCTGGCA		
ExPEC	*sfaS*	SfaS f	GTGGATACGACGATTACTGTG	240	Johnson & Stell [20]
		SfaS r	CCGCCAGCATTCCCTGTATTC		
ExPEC	*focG*	FocG f	CAGCACAGGCAGTGGATACGA	360	Johnson & Stell [20]
		FocG r	GAATGTCGCCTGCCCATTGCT		
ExPEC	*afa/draBC*	afa1	GCTGGGCAGCAAACTGATAACTCTC	750	Le Bouguenec *et al.* [23]
		afa2	CATCAAGCTGTTTGTTCGTCCGCCG		
ExPEC	*cnf1*	cnf1-f2	CAGGAGGTACTTAGCAGCGT	468	Mora *et al.* [22]
		cnf1-rc	TAATTTTGGGTTTGTATC		
ExPEC	*cdtB*	cdt-s1	GAAAGTAAATGGAATATAAATGTCCG	466	Tóth *et al.* [24]
		cdt-as1	AAATCACCAAGAATCATCCAGTTA		
		cdt-s2	GAAAATAAATGGAACACACATGTCCG		
		cdt-as2	AAATCTCCTGCAATCATCCAGTTA		
ExPEC	*sat*	SatF	GCAGCTACCGCAATAGGAGGT	937	Johnson *et al.* [25]
		SatR	CATTCAGAGTACCGGGGCCTA		
ExPEC	*hlyA*	hly F	AACAAGGATAAGCACTGTTCTGGCT	1177	Yamamoto *et al.* [26]
		hly R	ACCATATAAGCGGTCATTCCCGTCA		
ExPEC	*iucD*	Aer F	TACCGGATTGTCATATGCAGACCGT	602	Yamamoto *et al.* [26]
		Aer R	AATATCTTCCTCCAGTCCGGAGAAG		
ExPEC	*iroN*	Ironec-F	AAGTCAAAGCAGGGGTTGCCCG	665	Johnson *et al.* [27]
		Ironec-R	GACGCCGACATTAAGACGCAG		
ExPEC	*kpsM II*	KpsII f	GCGCATTTGCTGATACTGTTG	272	Johnson & Stell [20]
		KpsII r	CATCCAGACGATAAGCATGAGCA		
ExPEC	*kpsM II-K2*	KpsII f	GCGCATTTGCTGATACTGTTG	570	Johnson & O´bryan [28]
		KpsII-K2r	AGGTAGTTCAGACTCACACCT		

Pathotype	Target	Primers	Nucleotide sequence (5´- 3´)	Size (bp)	Reference
ExPEC	*kpsM II-K5*	K5-f	CAGTATCAGCAATCGTTCTGTA	159	Johnson & Stell [20]
		KpsII r	CATCCAGACGATAAGCATGAGCA		
ExPEC	*neuC*	neu1	AGGTGAAAAGCCTGGTAGTGTG	676	Moulin-Schouleur *et al.* [29]
		neu2	GGTGGTACATCCCGGGATGTC		
ExPEC	*kpsM III*	KpsIII f	TCCTCTTGCTACTATTCCCCCT	392	Johnson & Stell [20]
		KpsIII r	AGGCGTATCCATCCCTCCTAAC		
ExPEC	*cva*C	ColV-CF	CACACACAAACGGGAGCTGTT	680	Johnson & Stell [20]
		ColV-CR	CTTCCCGCAGCATAGTTCCAT		
ExPEC	*iss*	is-F	CAGCAACCCGAACCACTTGATG	323	Rodríguez-Siek *et al.* [30]
		is-R	AGCATTGCCAGAGCGGCAGAA		
ExPEC	*traT*	TraT f	GGTGTGGTGCGATGAGCACAG	290	Johnson & Stell [20]
		TraT r	CACGGTTCAGCCATCCCTGAG		
ExPEC	*ibeA*	ibe10 f	AGGCAGGTGTGCGCCGCGTAC	170	Johnson & Stell [20]
		ibe10 r	TGGTGCTCCGGCAAACCATGC		
ExPEC	*malX*	MALX-F	GCATGAGCAGTGCGATACATCGC	828	Mora *et al.* [22]
		MALX-R	AGGGCTGGGAAGTGGTTTAGCC		
ExPEC	*usp*	usp-F	ACATTCACGGCAAGCCTCAG	440	Bauer *et al.* [31]
		usp-R	AGCGAGTTCCTGGTGAAAGC		
ExPEC	*iutA*	aer-851F	GGCTGGACATCATGGGAACTGG	301	Johnson *et al.* [32]
		aer-1152R	CGTCGGGAACGGGTAGAATCG		
ExPEC	*tsh*	tsh03	GGTGGTGCACTGGAGTGG	640	Dozois *et al.* [33]
		tsh15	AGTCCAGCGTGATAGTGG		

[a]Primers used for the *eae* typing (amplification and sequencing).

Table S6. Primers used for the detection of TEM, SHV and CTX-M genes

Target	Primers	Nucleotide sequence (5´- 3´)	Size (bp)	Reference
bla_{CTX-M}	CTX-C3	ATGTGCAGCACCAGTAAAGTGATG	542	Mora *et al.* [22]
	CTX-C4	ACCGCGATATCGTTGGTGGTGCC		
bla_{SHV}	SHV-F2	TTGTCGCTTCTTTACTCGCC	879	Mora *et al.* [22]
	SHV-R2	CCCGGCGATTTGCTGATTTCGC		
bla_{TEM}	TEM-1-F	ATGAGTATTCAACATTTCCG	868	Rasheed *et al.* [34]
	TEM-1-R	CTGACAGTTACCAATGCTTA		

Figure S1. Phylogenetic tree based on the 489-nucleotide internal fragment of *fimH* (*fimH*41, *fimH*424, *fimH*438 and the new alleles) by the Neighbor-Joining method using MEGA6: numbers on the tree indicate bootstrap values calculated for 1,000 replicates

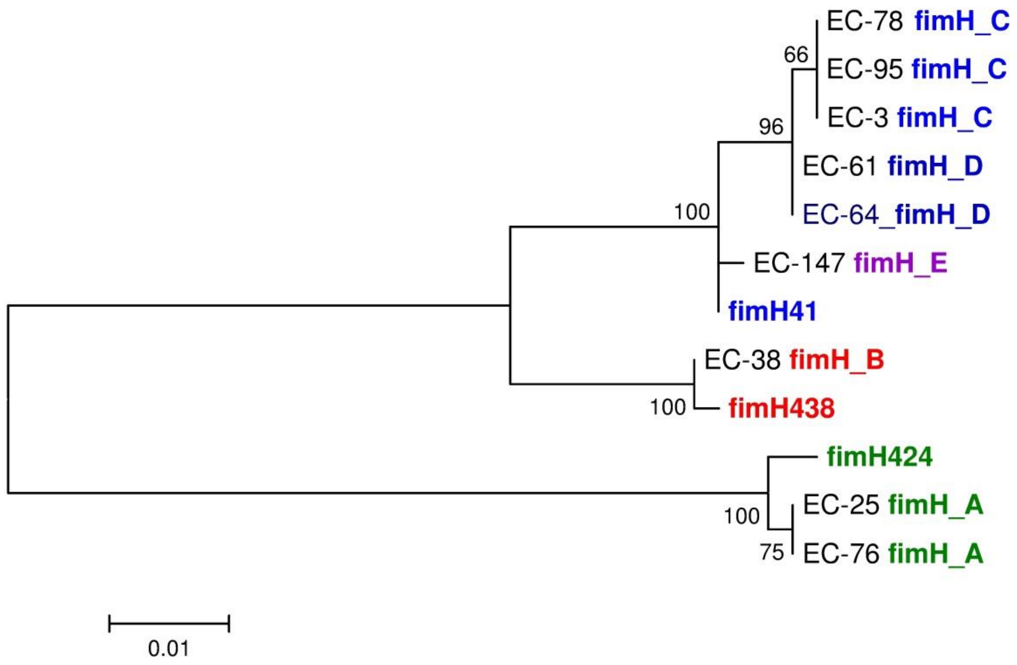

Figure S2. PFGE macrorrestriction profile of the *eae*-κ, *bfpA*-positive EC-147 isolate belonging to the clonal group O49:H10-A-ST206 (in blue) compared with human diarrheagenic isolates of the LREC collection, obtained from patients attending the Lucus Augusti Hospital (HULA) of Lugo, Spain: association between isolation code, serotype, ST, filogroup and origin is indicated on the right.

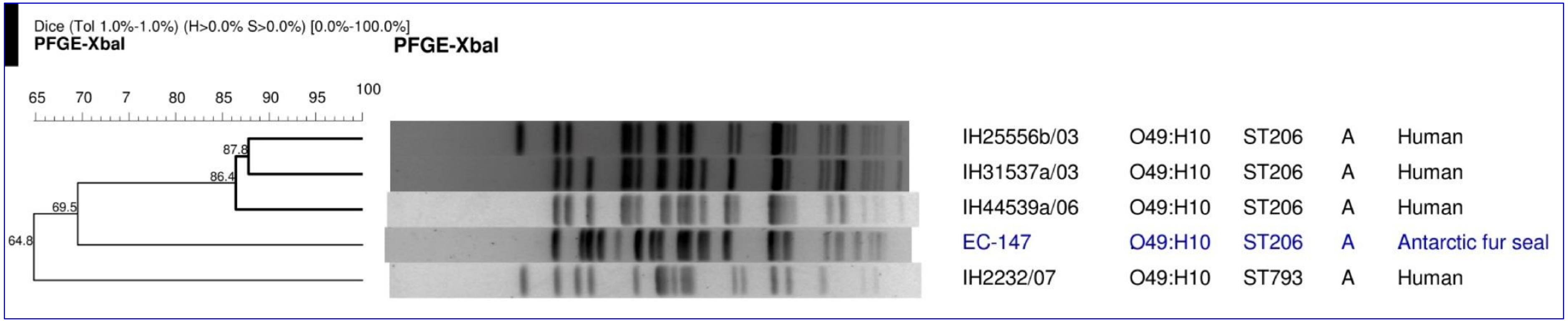

References

1. Clermont, O., Christenson, J.K., Denamur, E., & Gordon, M. The Clermont *Escherichia coli* phylo-typing method revisited: improvement of specificity and detection of new phylo-groups. *Environ. Microbiol. Reports*. **5**, 58-65 (2013).

2. Lindsey, R., García-Toledo, L., Fasulo, D., Gladney, L.M, & Strockbine, N. Multiplex poylmerase chain reaction for identification of *Escherichia coli*, *Escherichia albertii* and *Escherichia fergusonii*. *J. Microbiol. Methods*. **140**, 1-4 (2017).

3. Smati, M. *et al.* Quantitative analysis of commensal *Escherichia coli* populations reveals host-specific enterotypes at the intra-species level. *MicrobiologyOpen*. **4**, 604-6015 (2015).

4. Johnson, J. R. *et al.* Isolation and molecular characterization of nalidixic acid-resistant extraintestinal pathogenic *Escherichia coli* from retail chicken products. *Antimicrob. Agents Chemother*. **47**, 2161-2168 (2003).

5. Dahbi, G. *et al.* Molecular epidemiology and virulence of *Escherichia coli* O16:H5-ST131: Comparison with H30 and H30-Rx subclones of O25b:H4-ST131. *Int. J. Med. Microbiol*. **304**, 1247-1257 (2014).

6. CLSI. Vol. 26 ed. CLSI supplement M100S (Clinical and Laboratory Standards Institute, Wayne, PA, 2016).

7. Alonso, C. A. *et al*. Occurrence and characterization of stx and/or *eae*-positive Escherichia coli isolated from wildlife, including a typical EPEC strain from a wild boar. *Vet. Microbiol.* **207**, 69-73 (2017).

8. Mora, A. *et al*. Characteristics of the Shiga-toxin-producing enteroaggregative *Escherichia coli* O104:H4 German outbreak strain and of STEC strains isolated in Spain. International Microbiology 14, 121-141, doi:10.2436/20.1501.01.142 (2011).

9. Gannon, V. P. *et al*. Use of the flagellar H7 gene as a target in multiplex PCR assays and improved specificity in identification of enterohemorrhagic *Escherichia coli* strains. *J. Clin. Microbiol.* **35**, 656-662 (1997).

10. Mora, A. *et al.* Seropathotypes, Phylogroups, Stx Subtypes, and Intimin Types of Wildlife-Carried, Shiga Toxin-Producing *Escherichia coli* Strains with the Same

Characteristics as Human-Pathogenic Isolates. *App. Environ. Microbiol*. **78**, 2578-2585 (2012).

11. Durso, L. M., Bono, J. L. & Keen, J. E. Molecular serotyping of *Escherichia coli* O26:H11. *Appl. Environ. Microbiol.* **71**, 4941-4944 (2005).

12. Clermont, O. *et al*. The CTX-M-15-producing *Escherichia coli* diffusing clone belongs to a highly virulent B2 phylogenetic subgroup. *J. Antimicrob. Chemother*. **61**, 1024-1028 (2008).

13. Li, D. *et al.* A multiplex PCR method to detect 14 *Escherichia coli* serogroups associated with urinary tract infections. *J. Microbiol. Methods* **82**, 71-77 (2010).

14. Gunzburg, S. T., Tornieporth, N. G. & Riley, L. W. Identification of enteropathogenic *Escherichia coli* by PCR-based detection of the bundle-forming pilus gene. *J. Clin. Microbiol.* **33**, 1375-1377 (1995).

15. Tornieporth, N. G. *et al.* Differentiation of pathogenic *Escherichia coli* strains in Brazilian children by PCR. *J. Clin. Microbiol*. **33**, 1371-1374 (1995).

16. Schmidt, H. *et al*. Development of PCR for screening of enteroaggregative *Escherichia coli*. *J. Clin. Microbiol.* **33**, 701-705 (1995).

17. Schultsz, C. *et al*. Detection of enterotoxigenic *Escherichia coli* in stool samples by using nonradioactively labeled oligonucleotide DNA probes and PCR. *J. Clin. Microbiol.* **32**, 2393-2397 (1994).

18. Penteado, A. S. *et al.* Serobiotypes and virulence genes of *Escherichia coli* strains isolated from diarrheic and healthy rabbits in Brazil. *Vet. Microbiol.* **89**, 41-51 (2002).

19. Blanco, M. *et al*. Detection of *pap*, *sfa* and *afa* adhesin-encoding operons in uropathogenic *Escherichia coli* strains: relationship with expression of adhesins and production of toxins. *Res. Microbiol.* **148**, 745-755 (1997).

20. Johnson, J. R. & Stell, A. L. Extended virulence genotypes of *Escherichia coli* strains from patients with urosepsis in relation to phylogeny and host compromise. *J. Infect. Dis.* **181**, 261-272 (2000).

21. Marc, D. & Dho-Moulin, M. Analysis of the fim cluster of an avian O2 strain of *Escherichia coli*: serogroup-specific sites within *fimA* and nucleotide sequence of *fimI*. *J. Med. Microbiol.* **44**, 444-452 (1996).

22. Mora, A. *et al.* Poultry as reservoir for extraintestinal pathogenic *Escherichia coli* O45:K1:H7-B2-ST95 in humans. *Vet. Microbiol.* **167**, 506-512 (2013).

23. Le Bouguenec, C., Archambaud, M. & Labigne, A. Rapid and specific detection of the *pap*, *afa*, and *sfa* adhesin-encoding operons in uropathogenic *Escherichia coli* strains by polymerase chain reaction. *J. Clin. Microbiol*. **30**, 1189-1193 (1992).

24. Toth, I., Herault, F., Beutin, L. & Oswald, E. Production of cytolethal distending toxins by pathogenic *Escherichia coli* strains isolated from human and animal sources: establishment of the existence of a new cdt variant (Type IV). *J. Clin. Microbiol.* **41**, 4285-4291 (2003).

25. Johnson, J. R., Gajewski, A., Lesse, A. J. & Russo, T. A. Extraintestinal pathogenic *Escherichia coli* as a cause of invasive nonurinary infections. *J. Clin. Microbiol.* **41**, 5798-5802 (2003).

26. Yamamoto, S. et al. Detection of urovirulence factors in *Escherichia coli* by multiplex polymerase chain reaction. *FEMS Immunol. Med. Microbiol*. **12**, 85-90 (1995).

27. Johnson, J. R. *et al*. Molecular epidemiological and phylogenetic associations of two novel putative virulence genes, *iha* and *iroN* (*E. coli*), among *Escherichia coli* isolates from patients with urosepsis. *Infect. Immun*. **68**, 3040-3047 (2000).

28. Johnson, J. R. & O'Bryan, T. T. Detection of the *Escherichia coli* group 2 polysaccharide capsule synthesis Gene *kpsM* by a rapid and specific PCR-based assay. *J. Clin. Microbiol.* **42**, 1773-1776 (2004).

29. Moulin-Schouleur, M. *et al.* Common virulence factors and genetic relationships between O18:K1:H7 *Escherichia coli* isolates of human and avian origin. *J. Clin. Microbiol.* **44**, 3484-3492 (2006).

30. Rodriguez-Siek, K. E., Giddings, C. W., Doetkott, C., Johnson, T. J. & Nolan, L. K. Characterizing the APEC pathotype. *Vet. Res.* 36, 241-256 (2005).

31. Bauer, R. J. *et al.* Molecular epidemiology of 3 putative virulence genes for *Escherichia coli* urinary tract infection-*usp*, *iha*, and *iroN* (*E. coli*). *J. Infect. Dis.* **185**, 1521-1524 (2002).

32. Johnson, J. R. *et al.* Characteristics and prevalence within serogroup O4 of a J96-like clonal group of uropathogenic *Escherichia coli* O4:H5 containing the class I and class III alleles of *papG*. *Infect. Immun.* **65**, 2153-2159 (1997).

33. Dozois, C. M. *et al.* Relationship between the Tsh autotransporter and pathogenicity of avian *Escherichia coli* and localization and analysis of the Tsh genetic region. *Infect. Immun.* **68**, 4145-4154 (2000).

34. Rasheed, J. K. *et al.* Evolution of extended-spectrum beta-lactam resistance (SHV-8) in a strain of *Escherichia coli* during multiple episodes of bacteremia. *Antimicrob. Agents Chemother.* **41**, 647-653 (1997).
